# Drug-Induced Hematological Cytopenia in Kidney Transplantation and the Challenges It Poses for Kidney Transplant Physicians

**DOI:** 10.1155/2018/9429265

**Published:** 2018-08-01

**Authors:** Muhammad Abdul Mabood Khalil, Muhammad Ashhad Ullah Khalil, Taqi F. Taufeeq Khan, Jackson Tan

**Affiliations:** ^1^RIPAS Hospital, Bandar Seri Begawan BA1710, Brunei Darussalam; ^2^Institute of Kidney Diseases, Hayatabad, Peshawar 25000, KPK, Pakistan; ^3^King Salman Armed Forces Hospital, Tabuk King Abdul Aziz Rd., Tabuk 47512, Saudi Arabia

## Abstract

Drug-induced hematological cytopenia is common in kidney transplantation. Various cytopenia including leucopenia (neutropenia), thrombocytopenia, and anemia can occur in kidney transplant recipients. Persistent severe leucopenia or neutropenia can lead to opportunistic infections of various etiologies. On the contrary, reducing or stopping immunosuppressive medications in these events can provoke a rejection. Transplant clinicians are often faced with the delicate dilemma of balancing cytopenia and rejection from adjustments of immunosuppressive regimen. Differentials of drug-induced cytopenia are wide. Identification of culprit medication and subsequent modification is also challenging. In this review, we will discuss individual drug implicated in causing cytopenia and correlate it with corresponding literature evidence.

## 1. Introduction

Kidney transplantation is the optimal treatment for patients suffering from chronic kidney disease. It improves quality of life and survival. With the advent of new immunosuppressive medications for desensitization, induction, and maintenance, the incidence of rejection has reduced tremendously. However, these medications also have harmful and deleterious effects. Drugs which induced cytopenia are common in kidney transplant recipients (KTR). A delicate balance is needed to prevent rejection and avoid various complications including cytopenia. Cytopenia is common in kidney transplant recipients [1]. Twenty to sixty % of KTR will have one episode of either neutropenia or cytopenia during the course of their transplant [2]. Cytopenia is more common in the initial period due to induction therapy and intense maintenance immunosuppression. Similarly thrombocytopenia is also common in first year of transplantation and most KTR will have the lowest platelet levels within the first three months [2]. Various drugs have been implicated in cytopenia. These include mycophenolate mofetil (MMF) or enteric-coated mycophenolate sodium (EC-MPS) [3–5], ganciclovir/valganciclovir [3, 6, 7], antithymocyte globulin (ATG) [8, 9], tacrolimus [10, 11], sirolimus [12], and cotrimoxazole [13, 14]. Severe cytopenia warrants urgent intervention by identifying the culprit drug and reducing or stopping it. In such situations a balanced approach is needed. Stopping immunosuppressive medications can provoke rejection. Similarly holding valganciclovir or trimethoprim-sulphamethaxazole can predispose to cytomegalovirus or pneumocystis jirovecii infections. On the other hand, severe neutropenia or leucopenia can lead to life-threatening opportunistic infections. This review will focus on drugs implicated in hematological cytopenia and modification of drug or treatment regimen that can mitigate these complications.

## 2. Definition of Hematological Cytopenia

Various terminologies are used to define cytopenia and its severity. Pancytopenia refers to when all the three cell lines are affected. Bicytopenia refers to when 2 out of 3 cell lines are affected. Thrombocytopenia refers to low platelet count. The word leucopenia is often used interchangeably with neutropenia. A variety of definitions for leucopenia and neutropenia are available. Leucopenia is graded based on the Common Terminology Criteria for Adverse Events (CTCAE) [15]. CTCAE has graded leucopenia into 4 levels: grade 1 (lower range of normal limits to 3000 cells /mm^3^), grade 2 (2000-3000 WBC/mm^3^), grade 3 (1000-2000 WBC/mm^3^), and grade 4 (less than 1000 WBC/mm^3^). Most laboratories consider 4000 cells/mm^3^ as lower limit of normal and any level below this is considered as leucopenia. Others have used neutropenia to classify granulocytopenia according to its severity. They used Absolute Neutrophil Count (ANC) to assess the severity of neutropenia [16]. ANC is calculated as follows.

ANC = white blood cells (microliter) x percent (polymorphonuclear cells + bands)/100.

An ANC <1500/microliter or <1.5 x 109/L is defined as neutropenia and graded as mild, moderate, or severe [16]. In mild neutropenia, ANC will be in the range of 1000 to 1500/microliter or 1 to 1.5 x 109/L. Moderate neutropenia is defined as 500 to 999/microliter or 0.5 to 0.99 x 109/L. Severe neutropenia refers to ANC <500/microliter or <0.5 x 109/L.

CTCAE has graded thrombocytopenia into 4 levels [15]: grade 1 (75000 to 150,000 cells/mm3) grade 2 (50000-75000 cells/mm3), grade 3 (25000-50000 cells/mm3), and grade 4 (less than 25000/mm3). Platelet count of 150,000/mm3 is considered lower limit of normal in most laboratories.

## 3. Consequences of Hematological Cytopenia

Neutrophils and lymphocytes play important roles against infections. Leukopenic KTR are prone to develop opportunistic infections. Absolute neutrophil count less than 1000 cells per/L increases susceptibility for infections. Frequency and severity of infections are increased with decreasing neutrophil counts and prolonged duration of neutropenia [17, 18].* Escherichia coli* infections are more common in neutropenic KTR [19–21]. Neutropenic KTR has a higher incidence of intra-abdominal infection (22.5%) than a matched normopenic cohort (7-10%). Tacrolimus and MMF have often been associated with neutropenia [19]. Clinicians often reduce or stop MMF in the event of severe neutropenia. Although this is helpful in increasing white cell counts, it may lower threshold for rejection. This risk is especially prominent within the first year of transplant [20]. Zafrani L et al. [19] reported that high cumulative number of days without MPA was a strong predictor of acute rejection. Knoll et al. [22] also observed a decreased time to first acute rejection if the recommended dose of MPA was reduced from side effects. Similarly, Vanhove et al. [23] reported a significantly higher risk of rejections with greater than 50% dose reduction in MMF. While adjusting medications, baseline immunological status of KTR should be kept in mind. Those with high immunological risk and/or were transplanted within 1 year should be carefully monitored for rejection. By the same token, stopping prophylactic medications used for prevention of cytomegalovirus or pneumocystis jirovecii may provoke and precipitate these infections. In a nutshell, leucopenia increases the risk of infection by lowering the immunogenic threshold to ubiquitous and opportunistic pathogens.

## 4. Insight into Etiology of Hematological Cytopenia and Subsequent Modification of Individual Drugs

To identify the culprit drug for drug-induced hematological cytopenia is always challenging. This is because KTR is on multiple medications. Other than drugs, various other clinical conditions can cause drug-induced hematological cytopenia. Therefore it is important to take a detailed history and analyze sequence of event while looking for the cause of drug-induced cytopenia. For example majority of transplant medications including MMF, anti-CMV medications, and trimethoprim-sulphamethaxazole can cause leucopenia. Unfortunately, there is no evidence based approach to identify the culprit agent and to modify the medications. Most of decisions are based on clinical experience to tackle these situations. In this section various medications and the reported drug-induced cytopenia in the literature will be discussed.

### 4.1. Rituximab

Rituximab is a chimeric anti-CD20 monoclonal antibody, which leads to B cell depletion. After binding to CD-20 antigen, it depletes B cells through complement mediated cytotoxicity, phagocytosis by macrophages, and natural killer cells through antibody-dependent cell-mediated toxicity [24]. It is used in ABO-incompatible transplantation [25–27], treatment of acute graft rejection with B cell infiltrates [28], chronic antibody-mediated rejection [29, 30], and posttransplant lymphoproliferative disorder [31]. Rituximab grade 3 and 4 cytopenias were reported in 48% of patients. These included lymphopenia (40%), neutropenia (6%), leucopenia (4%), anemia (3%), and thrombocytopenia (2%) in lymphoma patients [32].

Rituximab has been reported to cause thrombocytopenia in many studies [33–41]. Several mechanisms for rituximab induced thrombocytopenia have been proposed. Some suggested that presence of CD20 antigen in the circulation causing antigen-antibody immune-mediated cell lysis by compliment activation [38, 39]. Others opine that platelets have CD-20 antigen on platelet surface [40]. Along the same vein, some postulate that anti-CD20/rituximab form soluble complex which binds platelet or it is due to intravascular fibrinolysis [41]. Most of the literature data reporting about thrombocytopenia stem from patients with hematological and autoimmune disorders. Rituximab induced thrombocytopenia is relatively rare in KTR. A randomized double-blind, placebo-controlled study of efficacy and safety of rituximab induction in KTR found no thrombocytopenia in rituximab arm [42]. Likewise in another prospective observational case control study, thrombocytopenia was not seen in KTR receiving rituximab [43]. However, some noncontrolled studies have reported thrombocytopenia in KTR who received rituximab [44, 45].

Rituximab can also cause neutropenia or leucopenia. Late-onset neutropenia (LON) is defined as a low neutrophil count occurring 4 weeks or more after rituximab treatment [37].LON is often reported in patients with lymphoma, chronic lymphocytic leukemia, and various autoimmune diseases treated with rituximab [37, 46–51]. The incidence of LON has been reported to be in the range of 4-27.3% [52, 53]. The reported median time to onset of LON is between 38 to 175 days and its duration varies from 5-77 days [54, 55]. It usually occurs after a median number of 6 doses of rituximab. LON has frequently been reported in KTR [44, 45, 56–59].The frequency of late-onset neutropenia in KTR has been reported from 37.5% to 48% in various studies [44, 45, 55, 56]. Common medications incriminated in causing LON include mycophenolate mofetil or anti CMV medications (ganciclovir / valganciclovir).Ahmadi F et al. [43] reported a high incidence (66.7%) of late-onset neutropenia [defined as neutropenia that occurred about 4 weeks after the last administered dose of rituximab without any other alternate explanation (e.g., unresponsive to dose reduction/cessation of ganciclovir/ valganciclovir, or mycophenolate mofetil)].

Some authors reported effect of rituximab on white cells as leucopenia in KTR [42, 60, 61]. The incidence of leucopenia due to rituximab has been reported to be in the range of 19%-24.6% in various studies [42, 61]. The relative risk of leucopenia is 8.8 when rituximab is used during induction in ABO-compatible nonsensitized renal transplantation [61].

Given that cytopenias are commonly reported in rituximab usage, transplant physicians have to be extra vigilant in monitoring the different lineages of blood, especially 4 weeks post-rituximab administration. The relevant drug doses can be either reduced or omitted to facilitate improvements of blood counts deficiencies. Rituximab induced thrombocytopenia rarely leads to bleeding [33, 62] and platelet infusions are debatable and usually not required. This can only be considered if low platelet count is complicated by bleeding [62, 63]. Specific therapy for neutropenia and thrombocytopenia has been discussed at the end.  Table 1 is showing summary of hematological complications caused by various medications used in KTR.

### 4.2. Antithymocyte Globulin (ATG)

Thymoglobulin is not specific for T-cells. It contains antibodies directed against different blood cell types (T-cells > B cells; NK cells > monocytes; neutrophils > platelets > erythrocytes) [68, 123]. Because of the presence of cross-reacting antibodies against nonlymphoid cells, hemolytic anemia, thrombosis, thrombocytopenia, and neutropenia can occur [68]. At high doses of thymoglobulin, nonspecific binding to neutrophils and platelets can lead to undesirable effects, such as transient neutropenia and thrombocytopenia [124–127]. The incidence of leucopenia is variable in KTR. This is largely due to inconsistency of duration and dosing regimens among users. Various authors reported incidence of leucopenia as 10% [64], 38% [65], 33.5% [66], and 50% [67].The high prevalence of 50% by Osama et al. could be due to concomitant use of azathioprine in the maintenance phase of immunosuppression [67]. The incidence of thrombocytopenia has been reported to be between 10% and 26.5% in KTR [64, 65]. Brennan reported that the major reasons for stopping or reducing the antithymocyte globulin dose were leucopenia (in 45.2% of patients), thrombocytopenia (11.9% of patients), or both (14.3% of patients) [66]. One should consider withholding ATG if the platelet count drops below 50,000 per mm^3^ or the white blood cell (WBC) count drops below 2,000 per mm^3^. One should consider halving the ATG dose [64, 69], if the platelet count is between 50,000 and 75,000 per mm^3^ or the WBC count is between 2,000 and 3,000 per mm^3^. Another effective way of avoiding cytopenia is to monitor CD3 (+) T-Cell. Keeping CD3 (+) T-Cell <0.05x109/L (<50/microliter) [128–131] has been a useful index to avoid excessive doses. Using this preemptive monitoring approach, early acute rejection, infectious episodes, and hematological complications, such as leucopenia and thrombocytopenia, are found to be less common [131].In places where this facility is not available total lymphocyte count can be used. Total lymphocyte count below 0.3 x 10(9)/l has been found to be a useful index for using ATG [129]. While treating ATG induced cytopenia, the effect of other immunosuppressive medications should be taken into consideration too. Mycophenolate mofetil (MMF) dose should be either reduced or stopped [65, 132] if ATG induced cytopenia has occurred. Care should be taken if mammalian target of rapamycin inhibitors are used with ATG as the combination has been known to induce thrombocytopenia [133]. Early steroids withdrawal after ATG induction is associated with high incidence of leucopenia [134, 135], as steroid myelostimulating effect is lacking.

### 4.3. Alemtuzumab

Alemtuzumab is a humanized monoclonal IgG1 antibody directed against CD52, a glycoprotein expressed on mononuclear cells, including T- and B-lymphocytes, monocytes, and natural killer cells [136, 137]. It has been used as an induction agent [138–140] and in treatment of acute rejection [141, 142].

The incidence of leucopenia in KTR has been reported as 33.3% and 42% in various studies [70, 71]. The combined incidence of leucopenia and neutropenia is 47% [72]. Alemtuzumab causes more myelosuppression than ATG [70, 71], with the lowest white cell count being observed approximately 130 days after the last administered dose [72]. However, alemtuzumab induced leucopenia rarely causes frequent or severe infections [71, 72, 143]. Alemtuzumab induced leucopenia usually results in the reduction of MMF dose and, as a result, the mean dose of MMF was 14 mg/kilogram which is significantly lower than those who received either ATG or daclizumab [143]. KTR with high immunological risk must be monitored for a potential risk of rejection while reducing MMF in such events [144]. Alemtuzumab has been incriminated in B cell dysregulation and causing autoimmune disorder. Autoimmune thrombocytopenia has been reported in multiple sclerosis and chronic lymphocytic leukemia, the incidence of which is 1-2.5% [74, 75]. Fatal cases of autoimmune thrombocytopenia has been reported [75]. Alemtuzumab induced thrombocytopenia has been reported in 14% of KTR in one cohort of patients [73]. Surgical bleeding necessitating reoperation occurred in 12% of patients, of which the majority had thrombocytopenia [73]. Bearing these factors in mind, the manufacturer recommends dose modification if absolute neutrophil count (ANC) is < 250/all and/or platelet count ≤25,000/all. For first occurrence of cytopenia, alemtuzumab therapy should be withheld. The therapy should be resumed at 30 mg when ANC ≥ 500/all and platelet count ≥ 50,000/all. For second occurrence, alemtuzumab therapy should be withheld and resumed when ANC ≥ 500/all and platelet count ≥ 50,000/all at a dose of 10mg. If cytopenia happened for a third time, then alemtuzumab should be discontinued indefinitely [145].

### 4.4. Interleukin Receptor Antagonist (IL2-R Antagonist)

IL2-R antagonists are monoclonal chimeric (basiliximab) and humanized (daclizumab) murine antibodies against CD25. They inhibit IL-2-mediated activation and proliferation of T-cells in transplant patients and are used as induction agents to prevent acute rejection [146]. Humanized (daclizumab) murine antibody has been withdrawn in the US and the rest of the world. Unlike ATG and alemtuzumab, IL2-R antagonists act only on activated T-cells. Therefore, leucopenia and thrombocytopenia are comparatively rare. Incidence of leucopenia and thrombocytopenia in basiliximab-treated renal transplant patients are approximately 10%–15% and 5%, respectively [76]. Many comparative studies have demonstrated that leucopenia and thrombocytopenia occurred less in basiliximab [64, 76, 141, 147, 148] when compared to other agents used in induction. In the 3 C trial, leucopenia was 3.6 times higher in KTR receiving alemtuzumab as compared to basiliximab [147]. Brennan et al. found leucopenia (in 33.3%) and thrombocytopenia (in 14.6%) in KTR who received ATG. In contrast leucopenia and thrombocytopenia were 10.6% and 5.8%, respectively, in the basiliximab group [149]. Another study showed that leucopenia and thrombocytopenia rates were significantly higher in the thymoglobulin group than in the basiliximab group (22.8% versus 11.8%, 8.1% versus 2.8%; P < .05) [77].

Like basiliximab, daclizumab has little effect on blood cells when compared to ATG or alemtuzumab. Leukocyte and platelet counts were higher in induction with daclizumab when compared to ATG [149]. In another randomized control trial, platelet and lymphocyte counts were significantly higher in daclizumab group when compared with ATG [78]. As a result of this mild effect of daclizumab, less modification is needed in MMF doses when compared to either ATG or alemtuzumab [143]. In fact the mean doses of MMF were higher in the daclizumab group when compared with KTR who received either ATG or alemtuzumab [143]. With these effects in mind, IL-2 R antagonist may be the preferred choice by many clinicians in low-to-intermediate immunological risk KTR, who have either leucopenia or thrombocytopenia.

### 4.5. Mycophenolate Mofetil (MMF) and Enteric-Coated Mycophenolate Sodium (EC-MPS)

MMF and EC-MPS are the inhibitors of inosine monophosphate dehydrogenase. They inhibits denovo pathway of guanosine nucleotide synthesis in T and B-lymphocytes and prevents their proliferation, thereby suppresses both cell-mediated and humoral immune responses [150, 151]. MMF has been shown to prevent acute graft rejection following renal transplantation [152, 153]. Mycophenolate-related leucopenia occurs in 11.8% to 40% of KTR [79–82]. The hematologic complication as a result of marrow suppression is the most frequent reason for MMF dose reduction [23]. Around 245 (46.5%) of the reduction events were due to leucopenia (n = 178), anemia (n = 22), thrombocytopenia (n = 19), and pancytopenia (n = 40) [23]. The myelosuppressive effects of MMF are dose-dependent and correlate with trough levels of the active metabolite, mycophenolic acid (MPA) [2, 154]. Use of other concurrent medications can also contribute. Valganciclovir [3], valaciclovir [155], and fenofibrate [156] may exacerbate MMF induced leucopenia. As discussed previously, ATG and alemtuzumab induced cytopenia may cloud diagnosis and often lead to MMF dose reduction, whether or not the latter had been the primarily incriminated [143]. Genetics may also play a role in mycophenolate-related hematologic toxicity and single nucleotide polymorphism has been implicated in MMF induced cytopenia [157]. MMF induced neutropenia or leucopenia requires dose reduction or omission of the drug completely [23, 154, 158]. Dose reduction has been associated with increased risk of acute rejection and graft loss in several retrospective studies. Being retrospective in nature, many of these studies were flawed due to many confounding factors including immunological risk assessment which can affect occurrence of rejection [20, 22, 159]. Most MMF dose reduction occurs in the first year after transplant, during which the KTR is at the highest of risk of rejection [159]. There are several ways to approach leucopenia after transplantation while minimizing reduction or discontinuation of immunosuppressive medication. Preemptive reduction in dosing of MMF with ATG or alemtuzumab induction with careful monitoring may be one option. Reduction of prophylactic valganciclovir dose by 50% against cytomegalovirus may be preventative too. Valganciclovir in a dose of 450 mg once a day has been shown to be equally efficacious in preventing cytomegalovirus infection when compared with 900 mg once a day [160]. Reducing or holding MMF and valganciclovir is useful in reverting these blood cytopenia [71]. Usage of mTOR inhibitors like sirolimus or everolimus may also help to avert leukopenic complications, providing there is no contraindication to its use [5, 161].

### 4.6. Calcineurin Inhibitors: Tacrolimus and Cyclosporine

Calcineurin inhibitors include cyclosporine and tacrolimus. They are useful in prevention of acute rejections. Tacrolimus is considered more potent and reduces rejection rate and results in better graft survival at 1 year [162]. Hematologic abnormalities have been reported more frequently with tacrolimus. In one study out of 65 hematologic abnormalities, 11 (16.92%) were attributed to tacrolimus in a cohort of cardiothoracic transplantation [10]. These eleven episodes included anemia (7/11), neutropenia (1/11) and simultaneous anemia and neutropenia (3/11). Tacrolimus potentiates myelosuppressive effects of MMF. This combination causes neutropenia in 28% of KTR [19]. There are various proposed mechanisms for tacrolimus induced neutropenia or leucopenia. Some believe that tacrolimus may cause direct inhibitions of myeloid cells. Bone marrow hypoplasia due to tacrolimus has been reported in liver transplant [163]. However, direct myeloid inhibition was not found in in vitro studies [164, 165]. Bone marrow examination in in vivo case series failed to document myeloid maturation arrest [11]. Therefore, direct inhibition of myeloid precursors may not explain completely the mechanism of tacrolimus induced neutropenia or leucopenia. Another possible explanation is thought to be alternation of cytokines productions by T lymphocytes and monocytes. However use of antibodies against cytokines failed to show any difference in enhancement of myeloid progenitor cell colony forming units [164]. Autoantibodies against myeloid precursors or mature neutrophils is another mechanism that may explain tacrolimus induced cytopenia. Paradoxically, tacrolimus has been used as a drug for autoimmune disorders and this antagonizes the theory of marrow autoimmunity. Tacrolimus inhibits glucuronidation of mycophenolic acid (MPA) leading to its increased blood level [166, 167]. At the same time unlike cyclosporine, tacrolimus does not affect enterohepatic circulation of MMF leading to increase MPA level [168]. Therefore through inhibition of glucuronidation of MPA and uninterrupted enterohepatic recirculation, tacrolimus can result in higher MPA levels which can lead to marrow toxicity [169]. Calcineurin inhibitors may also indirectly cause thrombocytopenia through thrombotic microangiopathy [83–87].

Tacrolimus induced neutropenia usually occurs within first three months [11]. Cytopenia in early transplant is multifactorial and there is no diagnostic test available to diagnose tacrolimus induced neutropenia. Definitive proof requires cessation of the drug and normalization of white cell count [11]. Tacrolimus administration along with MMF increases area under curve for MMF gradually over 3 month by 20-30% [170]. Keeping these studies in mind, many studies conducted in Asian renal transplant recipients, including a randomized controlled trial, suggested the need for MMF dose reduction in patients with tacrolimus to minimize the side effects of MMF including myelotoxicity [171–173]. If calcineurin inhibitor induced thrombotic microangiopathy occurred, everolimus, belatacept, or eculizumab can be considered as alternative options [84, 86, 87].

### 4.7. Azathioprine

Azathioprine (AZA) is an inhibitor of purine synthesis and has been used as an immunosuppressant since the 1960 [174]. MMF has replaced AZA as the preferred antimetabolite agent in kidney transplantation over the past few decades. Pooled efficacy analysis of these trials demonstrated a significant reduction in acute rejection rates with use of MMF compared to AZA or placebo [175]. AZA causes leucopenia/neutropenia in around 50% of KTR [88]. Frequency of leucopenia increases significantly following azathioprine dosage exceeding 1.99 mg/kg body weight/day [89]. AZA induced leucopenia occurs earlier, usually during the first 5 weeks of transplantation. Most leucopenia settles after reducing the dose or temporary cessation of the drug [89]. Repeat leukopenic incident occurred in 70% of patients who previously had a drug leukopenic event [89]. Azathioprine can cause macrocytosis and megaloblastic changes in bone marrow, which in turn can lead to ineffective erythropoiesis and pancytopenia [90–92]. Azathioprine is metabolized to 6-mercaptopurine (6-MP). 6-MP is catalyzed by the enzyme thiopurine S-methyltransferase (TPMT), to form the inactive metabolite. TPMT activity is controlled by a genetic polymorphism [176–178]. Patients with intermediate thiopurine S-methyl transferase (TPMT) activity may be at an increased risk of myelotoxicity if receiving conventional doses of AZA. Patients with low or absent TPMT activity are at an increased risk of developing severe, life-threatening myelotoxicity if receiving conventional doses of AZA. To decrease myelosuppression, various strategies have been proposed. Monitoring with red blood cell 6-thioguanine nucleotide is more useful when compared with plasma 6-mercaptopurine [179]. TPMT genotyping or phenotyping can help identify patients who are at an increased risk of developing AZA toxicity [180–187]. Allopurinol has an important interaction with AZA causing reduction in the metabolism of purines to uric acid by inhibiting the activity of the enzyme xanthine oxidase. If allopurinol is used, AZA dosage should be reduced by 25-50% to avoid myelosuppression [188, 189].

AZA is preferred to MMF in areas where cost of medication is an issue [190]. Like, the other drugs, vigilance for myelotoxicity is important and it is suggested that patients on AZA should have complete blood counts, including platelet counts, weekly during the first month, twice monthly for the second and third months of treatment, then monthly or more frequently if dosage alterations or other therapy changes are necessary [191].

### 4.8. Mammalian Target of Rapamycin Inhibitors (MTORi)

Sirolimus and everolimus are the two most commonly used MTORi in kidney transplantation. Bone marrow toxicity leading to cytopenia is a well-known phenomenon of MTORi [12, 192–194]. Post-transplant anemia occurs in 12% to 76% of KTR and MTOR inhibitor is one of the frequent causes among these etiologies [194]. Anemia usually appears within the first month of initiation of MTOR inhibitor and it persists throughout its course of therapy [93]. The combination of MMF and MTORi is associated with more anemia when compared to other combinations [94, 195–197]. Disturbed iron metabolism, impaired iron absorption, and early maturation of erythroid precursor leading to decrease globulin synthesis are various proposed mechanisms [94, 95]. Assessing iron status in these patients is important and iron replacement and erythropoietin can often correct these complications [95, 196].

In a meta-analysis of eight trials where calcineurin inhibitors were replaced with MTORi, the latter caused significant bone marrow toxicity resulting in leucopenia and thrombocytopenia [96]. Bone marrow toxicity occurred in a dose-dependent fashion [198, 199] and occurred in 20% of KTR receiving sirolimus [12]. Various studies on sirolimus have reported that trough level greater than 12 to 16 microgram l^−1^ has an association with leucopenia and thrombocytopenia [12, 200]. Hematological manifestation is more common in first 4-8 weeks [12, 201]. Eighty-nine percent of sirolimus induced cytopenia resolves completely. Seven percent resolves with dose reduction and 4 percent requires temporary cessation of the drug [12]. These effects are more pronounced when MMF and MTORi are used simultaneously [202–205]. Induction with alemtuzumab followed by sirolimus and MMF combination in a steroid and CNI free regimen also lead to significant leucopenia [206].

Like sirolimus, everolimus has also been associated with hematological toxicities leading to leucopenia and thrombocytopenia [96, 97, 194, 199, 207]. The incidence of leucopenia and thrombocytopenia with everolimus is 11-19% and 10-17%, respectively [97].

Various proposed mechanisms of MTORi myelotoxicity have been reported. Sirolimus causes increase platelet aggregations and degranulation in response to adenosine monophosphate and thrombin in in vitro setting. MTORi inhibits signal transduction via the gp130 [beta] chain. A variety of cytokines including interleukin-11 [208], granulocyte colony stimulation factor, and erythropoietin through signal transduction via the gp130 [beta] chain stimulate production of erythrocytes, leukocytes, and platelets [209]. Therefore MTORi through inhibition of signal transduction via the gp130 [beta] chain may lead to various cytopenia. In addition, MTORi may cause anemia and thrombocytopenia indirectly by causing thrombotic microangiopathy. Both everolimus [98–100] and sirolimus [101, 102] have been shown to cause thrombotic microangiopathy.

MTORi induced cytopenia usually occurs after shifting KTR from steroids, CNI, and MMF combination to steroids, MTORi, and MMF. Most leucopenia resolves completely [12]. In those with persistent leucopenia, MMF dose may be reduced and MTORi doses should be adjusted to the lower end of therapeutic range [207]. Occasionally refractory cases may lead to discontinuation to MTORi [201, 206].

### 4.9. Valganciclovir

Valganciclovir is a valyl-ester prodrugs of oral ganciclovir. It has a bioavailability of nearly 70 % (compared with 7% for oral ganciclovir). Because of increased bioavailability, myelotoxicity is greater compared to ganciclovir and non-ganciclovir medications [106, 110]. The incidence of leucopenia is 10-28% in various studies in KTR [3, 103, 106–108]. Neutropenia has been reported in various studies from 4.9% to 37.5% in various studies [3, 103–105]. Many factors can affect prevalence and severity of these cytopenia. Prolonged prophylaxis of 200 days with valganciclovir causes more neutropenia than shorter period of prophylaxis (100 days) (38% versus 26%) [108]. Use of higher dose (900 mg) was significantly associated with occurrence of leucopenia [3] and neutropenia [110]. Patients with lower body mass index are also associated with significant leucopenia [105]. Concurrent use of MMF also potentiates valganciclovir myelotoxicity [3, 210]. Leucopenia with valganciclovir occurs within 3 months [3], with the majority resolving with or without treatment and with low risk of incidental infections [3, 106]. The need for granulocyte colony stimulating factor is higher in patients with longer duration of prophylaxis (14% versus 13%) [108]. If neutropenia occurs, conventional management will advise the reduction of dose of valganciclovir to 450 mg once a day or temporary omission [3]. Since low dose (450 mg once a day) has been shown to be equally efficacious in preventing cytomegalovirus infection when compared to high dose (900mg once a day) [160], it may be advisable to use low-dose prophylaxis to prevent myelosuppression.

### 4.10. Ganciclovir

Ganciclovir is used in the treatment and prevention of CMV infection in KTR. Ganciclovir can be given by intravenous route or via oral route. However, since oral ganciclovir has poor bioavailability, relatively high doses are needed (1000mg three times a day). Ganciclovir can cause myelosuppression leading to leucopenia, thrombocytopenia, and anemia [106, 211]. The incidence of leucopenia is 7.1% to 23.1% in various studies [106, 109]. The incidence of thrombocytopenia and anemia was 23.1% and 38.5% as reported in one study [106]. Generally speaking, the myelosuppressive effects of ganciclovir are modest when compared to valganciclovir. This is because valganciclovir has ten times more bioavailability than ganciclovir. As a result, the risk of neutropenia in valganciclovir is 188% higher than ganciclovir [110]. Paya C et al. reported less leucopenia (7.1% versus 13.5%) and neutropenia (3.2% versus 8.2%) with ganciclovir when compared with valganciclovir [106]. Majority (23%) of ganciclovir induced cytopenia improved with dose reduction [109]. Withholding of ganciclovir for cytopenia was reported is only in 2.4% of cases [106].

### 4.11. Valaciclovir

Valaciclovir has also been used with success in preventing CMV infection in KTR [111]. Bone marrow toxicity with valaciclovir is mild as compared to valganciclovir or ganciclovir. In a randomized control trial it was associated with anemia in 11-14% and leucopenia in 6-14%. However more importantly this finding was not significant when compared to placebo [111]. The risk of neutropenia is also less with valaciclovir. The risk of neutropenia with valganciclovir is 730% higher than valaciclovir [110]. MMF in combination with valaciclovir may lead to more myelotoxicity [109]. It has been suggested that MMF may lead to more intracellular concentration of valaciclovir leading to myelotoxicity [155]. As a result of lesser myelotoxicity, dose modifications of valaciclovir are less frequent. Discontinuation of ganciclovir is higher when compared with valaciclovir (23.1% versus 8.3%) in cases of cytopenic complications [109]. However, due to the high doses used (2 gram 4 hourly), the pill burden is very high and side effects like neurological symptoms occur more commonly [111, 212].

### 4.12. Trimethoprim-Sulphamethaxazole (TMP-SMZ)

TMP-SMZ is used for prophylaxis of pneumocystis jirovecii. It is well known for causing blood cytopenia including neutropenia/leucopenia, thrombocytopenia, and megaloblastic anemia. Inhibition of hematopoiesis has been shown in many in vitro studies. Trimethoprim inhibits granulopoiesis and erythropoiesis in vitro in a dose-dependent fashion [112]. The effects are reversed with folinic acid supplementation. Similar effects were demonstrated on folate depleted granulocyte precursors in another study done in vitro setting [213]. It is proposed that the effect of large doses of trimethoprim on the hematopoietic system is probably the result of interference with the methylation of deoxyuridine arising from inhibition of dihydrofolate reductase, specifically in presence of folate deficiency. As a result TMP-SMZ also causes megaloblastic changes both in peripheral blood and in bone marrow especially if there is deficiency of folate [214, 215]. Folinic acid supplementation in case of folate deficiency may be helpful in these patients.

Analysis of the Swedish reporting system over a period of 10 years reported 154 blood cytopenia due to TMP-SMZ, out of which 39.61% (61 / 154) were leucopenia and 18.18% (28/154) were thrombocytopenia. Among leukopenic patients, 16 (10.38%) were neutropenic [13]. Neutropenia usually occurred early with initiation of therapy usually within 10 days [216, 217].

TMP-SMZ associated blood cytopenia has also been studied in KTR. TMP-SMZ is safe to use in KTR and one randomized control trial showed no evidence of myelosuppression [218]. However the low dose of azathioprine and folic acid supplementation in that trial may have led to low incidence of myelotoxicity. Chemoprophylaxis with TMP-SMZ for* Pneumocystis jirovecii* in KTR causes leucopenia in 2 % of KTR. Combination of azathioprine and TMP-SMZ compared to azathioprine alone in KTR was associated with more myelotoxicity [219]. It was shown in bone marrow culture that TMP-SMZ enhanced marrow suppressive effect of mercaptopurine in KTR [220]. Rarely TMP-SMZ may cause drug-induced thrombocytopenia in KTR [221].

### 4.13. Dapsone

Dapsone is often used as a second-line agent for* Pneumocystis jirovecii* prophylaxis and can cause a variety of hematological manifestations [222]. Dapsone induced neutropenia has been reported in literature [113, 114] and it can be severe enough to result in agranulocytosis [115, 116]. Dapsone can also result in acquired methemoglobinemia [117–121]. Methemoglobinemia occurs due to accumulation of N-hydroxylated metabolites [223] and has been reported in KTR [122]. The incidence of dapsone induced methemoglobinemia in KTR is 46% and in the majority of the cases (50%) remains asymptomatic [122]. Dapsone, tacrolimus, and sirolimus are all metabolized by P-450 isoenzyme, CYP3A4. Administration of these medications with dapsone may increase N-hydroxylated metabolites resulting in more methemoglobinemia [223]. As a result, one has to be vigilant for hemolysis from methemoglobinemia in patient taking dapsone. Alternative agents like pentamidine can be used as prophylaxis with careful monitoring.

## 5. Differential Diagnosis of Drug-Induced Cytopenia

Blood cytopenia has a wide range of etiologies. Therefore, it is important to keep these in mind while managing these patients. Varieties of conditions can cause post-transplant anemia other than medications. Allograft dysfunction with subsequent reduced erythropoietin production is one of the major causes of post-transplant anemia [224]. Various viruses including parvovirus B19 (PVB19), cytomegalovirus (CMV), and Epstein-Barr virus (EBV) in KTR can cause aplastic anemia [2]. Parvovirus has been reported to cause pure red cell aplasia in KTR [225]. Acute rejection can cause post-transplant anemia due to decreased erythropoietin production and disturb the binding and transport of iron and folate through systemic inflammatory response [226]. Similarly iron deficiency anemia has been incriminated to be one of the causes of post-transplant anemia [227]. Hemolytic anemia can occur in ABO-incompatible transplantation. Hemolysis following ABO-incompatible transplantation is caused by a type of graft-versus-host reaction in which the B-lymphocytes in the donor organ produce ABO antibodies to the ABO antigens of the recipient [228].

Similarly, leucopenia and thrombocytopenia can be caused due to many other etiologies other than drugs. Like general population B12, folic acid, zinc, and copper deficiency may lead to leucopenia/neutropenia in KTR [229]. Post-Transplant Lymphoproliferative Disorder (PTLD) due to EBV infection should also be kept in mind as a differential [229–231]. PTLD can involve lymphatic system and various organs of the bodies. It can cause cytopenia by infiltrating the bone marrow [231]. Various viruses including CMV, PVB19, herpesvirus-6 (HHV-6), influenza, and ehrlichiosis can cause myelosuppression which can lead to cytopenia [232]. Hemophagocytic Syndrome (HPS) can also present with cytopenia. It is caused by various opportunistic viral infections such as CMV, adenovirus, EBV, human herpes virus 8 (HHV-8), human herpes virus 6 (HHV-6), PVB19, and BK polyoma virus [233]. Underlying etiology of HPS should be searched for and treated in time as HSP can be fatal if missed [234]. Other than drugs a variety of conditions can cause thrombotic microangiopathy. Viral infections (CMV, HIV, and PVB19), severe renal ischemia, and antibody-mediated acute humoral rejection have been implicated in thrombotic microangiopathy [2]. Thrombotic microangiopathy manifests as thrombocytopenia, thrombosis in blood vessels leading to graft dysfunction and fragmentation of red blood cells.

## 6. Specific Therapy for Neutropenia and Thrombocytopenia

### 6.1. Neutropenia

As previously discussed, many medications used in KTR can lead to neutropenia. Absolute Neutrophil Counts (ANC) can be used to assess the severity of neutropenia. Severe neutropenia with ANC <500/microliter or <0.5 x 109/L is associated with pneumonia, sepsis, and septicemic shock [235]. Worsening neutropenia (absolute neutrophil count [ANC] <100 cells/mm^3^ of prolonged duration (7 days) is considered to be at high risk of getting an infection [236].

Detailed history and sequence of events may identify the culprit drug. Unfortunately there is no diagnostic test or evidence based approach to identify the culprit medication. Definite diagnosis requires reduction or cessation of drug and normalization of cell counts. If neutropenia persists despite modification of immunosuppressive medications, then one may opt for colony-stimulating factors to increase white cell counts. Stimulation of innate immunity by increasing expression of cytokines may have consequences. Activation of innate immunity may lead to activation of adaptive immunity which may lead to graft injury [237].

Colony-stimulating factors have been used in KTR with leucopenia [71]. Granulocyte Colony-Stimulating Factor (G-CSF) leads to proliferation of neutrophils and reduced production of inflammatory cytokines including tumor necrosis factor, interleukin-1, interleukin-12, and interferon. At the same time, it increases anti-inflammatory soluble TNF receptors p55 and p75, as well as IL-l receptor antagonist (lL-lra) and prostaglandin E2 [238–241]. There is no G-CSF receptor on lymphocyte and it has minimal effect on lymphocytes [239]. Few studies suggest that G-CSF may actually lower rejection rates [242, 243]. Various studies are done on G-CSF in kidney and liver transplants with variable beneficial effects. Analysis of various studies showed that G-CSF improves white cell count, reduces infections, and does not provoke rejections [244–252]. However a randomized control trial in liver transplant by Winston and his colleagues showed no beneficial effects on infection, rejection, or survival. This is despite producing substantial increase in white blood cells [253].

Granulocyte-Monocyte Colony-Stimulating Factor (GM-CSF) is another stimulating agent which activates neutrophil, monocytes, macrophages, and dendritic cells and it has a proinflammatory profile unlike G-CSF [254–257]. Data on GMC-CSF use in KTR has been scanty. It was found safe in various studies in improving white cell counts and reducing infections in kidney, liver, and heart transplant [258–262]. However, there is no randomized trial done on GM-CSF to assess its benefits and theoretical risk of rejection. A review by Page and his colleagues on G-CSF and GM-CSF concluded that there is a need for further studies for use of these agents in solid organ transplantation [263].  Table 2 is showing summary of studies on G-CSF and GM-CSF done in solid organ transplantation.

In summary G-CSF and GM-CSF are safe to be used in kidney transplantation. However their benefits in prevention of infection in KTR require further evaluation. The most practical approach in dealing with neutropenia is to identify potential culprit agent/s. MMF, valganciclovir, ganciclovir, TMP-SMZ, and agents used in inductions (alemtuzumab and ATG) can all cause neutropenia, either in isolation or as a combination. Most clinicians reduce MMF or temporarily stop it. Similarly, other agents like valganciclovir and TMP-SMZ can also be withheld until recovery of white cell counts. One should watch out for opportunistic infections and be wary of the theoretical risk of rejection. In the majority of the cases, white cell counts will recover if the suspect culprit drugs are identified and withheld or reduced. There are no clear guidelines for using either G-CSF or GM-CSF in drug-induced cytopenia in context of solid organ transplantation. Much of the evidence for use of these agents is available from the use of these agents in oncology. These agents are used for primary prophylaxis if risk of febrile neutropenia is greater than 20%, based on regimen of chemotherapy used or special situations [264]. Special situations include reduced bone marrow reserves (e.g., ANC <1.5 × 109/l) due to radiotherapy of >20% marrow, human immunodeficiency virus, and elderly patients older than 65 years treated with curative intention. It is used as therapy in patients with febrile neutropenia >7 days, hypotension, sepsis, pneumonia, or fungal infection [264]. Current evidence suggests that prolonged neutropenia of greater than 7 days [236], presence of fever, and severe neutropenia with ANC count less than 500/microliter [235] are bad prognostic factors and warrant the use of these agents prophylactically to prevent infection. Additionally, it should also be considered as adjunctive therapeutic agents along with antimicrobials, if there is febrile neutropenia >7 days, hypotension, sepsis, pneumonia, or fungal infection [264].

### 6.2. Thrombocytopenia

Thrombocytopenia in kidney transplantation is due to either myelosuppression or idiosyncratic immune reaction. A thorough evaluation of drugs is needed to find the culprit agents. Drug-induced thrombocytopenia is abrupt and may cause bleeding [265, 266]. Reducing the dose or withholding the drug in case of myelosuppression will lead to normalization of platelet counts. Sudden onset idiosyncratic drug-induced thrombocytopenia (for example with TMP-SMZ) will require the immediate cessation of the offending medication [267–270].

The majority of the cases will resolve with reduction of the dose or stopping the medications. Platelet transfusion is rarely required except if there is a high bleeding tendency or levels are exceptionally low (less than 10,000/ microliter). Sometimes it may also be necessary to transfuse if there is a pending invasive investigation like biopsy. There are no guidelines for platelet transfusion in solid organ transplantation and most of the evidence for platelet transfusion is extrapolated from literature involving hematological disorders. A study involving acute leukemia patients found that major bleeding occurred in only 0.8% of the days when platelet counts are between of 20,000 -50,000/microliter and on 0.07% on days when platelet count exceeded 100,000/microliter. They further suggested that gross hemorrhage rarely occurred at platelet count greater than 20,000/microliter [271]. In a randomized trial, the need for transfusion at 10,000 versus 20,000/microliter was evaluated and the authors found no significant statistical difference in bleeding between two groups [272]. Another study showed that the risk of bleeding was 21.5% in leukemic patient having a platelet count of 10,000/microliter as compared to 20% in those with 20,000/microliter [273]. Other studies also found a platelet count 10,000/microliter as a cut-off for prophylactic platelet transfusion to prevent bleeding [274–276]. Cut-off for therapeutic platelet transfusion for invasive procedures including gastroscopy and biopsy, insertion of indwelling lines, transbronchial biopsy, liver biopsy, laparotomy, or similar procedures has been greater than 50,000/microliter [277–279]. For ocular and neurosurgical procedure platelet count of ≥ 100,000 / ul is recommended [278]. For lumbar puncture recommended platelet count is ≥ 50,000 /ul [280]. Kidney is a vascular organ and most nephrologists recommend platelet count ≥ 100,000/ul [281]. Minor bleed and anemia (low hematocrit) may predict major bleed in thrombocytopenic patients [282, 283] and may warrant platelet transfusion even at high platelet count than 100,000/microliter. Theoretically transmission of cytomegalovirus through platelets is rare, but presence of associated occasional leukocytes in platelet concentrate may transmit CMV to the recipient [284].  Table 3 shows guidelines for platelet transfusion.

Thrombopoietin receptor agonist, romiplostim, and eltrombopag have been successfully used for treatment of thrombocytopenia in chronic idiopathic thrombocytopenic purpura in various randomized control trials [285, 286]. Moreover eltrombopag has been successfully used in thrombocytopenia in patients infected with hepatitis C virus infection [287] and aplastic anemia [288]. There is some evidence advocating the use of romiplostim and eltrombopag for chemotherapy induced thrombocytopenia [289–292]. However further studies are needed for use of these agents in this setting. There is minimal experience of these agents in context of drug-induced thrombocytopenia in kidney transplantation. In our literature search we came across a retrospective analysis of tacrolimus induced thrombocytopenia where various agents were used including romiplostim. They failed to improve platelet count [293]. Yet in another case report eltrombopag and plasmapheresis were successfully used as rescue therapy of acute post-renal transplant immune thrombocytopenia in a child with Schimke immuno-osseous dysplasia [294]. Further evidence is needed for its usefulness and safety in drug-induced thrombocytopenia in context of kidney transplantation.

## 7. Conclusions

Drug-induced blood cytopenia is common in kidney transplantation. The incidence is higher in the first year when immunosuppression is intense. Drug-induced leucopenia or neutropenia increases risk of infections. Dose reduction or withholding of culprit immunosuppressive drugs or prophylactic antimicrobials or antiviral increases theoretically may increase risk of rejection or opportunistic infections. There is no diagnostic test or evidence based approach to find the culprit medication. Dose reduction or cessation with normalization of blood cell counts identifies the offending agent. The majority of the cases can be improved through simple modification of doses or temporary cessation of medication. There is paucity in robust data for the use of G-CSF and GM-CSF in treatment of neutropenia. However selected cases with severe neutropenia (< 500 /ul) of greater than 7 days duration and fever may achieve benefit. There is not enough evidence to advocate the use of thrombopoietic agents in the treatment of thrombocytopenia in KTR.

## Figures and Tables

**Table 1:** Summary of drug-induced hematological cytopenia.

Drugs	Hematological cytopenia	Management
Rituximab	It causes late onset neutropenia in 37.5% to 48% [44, 45, 55, 56], leucopenia in 19%-24.6[42, 61], anemia in 3% [32], lymphopenia in 40% [32] and rarely thrombocytopenia [44, 45].	Late onset neutropenia is diagnosis of exclusion. It is suggested to reduce doses of anti CMV medication and MMF in face of neutropenia. In case of persisting neutropenia further doses of rituximab may be avoided.

ATG	It causes leucopenia in 10%-50% [64–67], hemolytic anemia [68]and thrombocytopenia in 10%-26.5% [64, 65].	Monitor CD3 subset or absolute lymphocyte count. Consider withholding ATG if the platelet count drops below 50,000 per mm^3^ or the white blood cell (WBC) count drops below 2,000 per mm^3^. Also consider halving the ATG dose [64, 69], if the platelet count is between 50,000–75,000 per mm^3^ or the WBC count is between 2,000–3,000 per mm^3^. Consider using reduce dose of MMF during ATG administration.

Alemtuzumab	It causes leucopenia in 33.3-42% [70, 71], combined incidence of leucopenia and neutropenia in 47% [72], transient thrombocytopenia 14% [73] and autoimmune thrombocytopenia in 1-2.5% [74, 75].	Consider dose modification if absolute neutrophil count (ANC) is < 250/all and/or platelet count ≤25,000/all. For first occurrence of cytopenia, alemtuzumab therapy should be withheld. The therapy should be resumed at 30 mg when ANC ≥ 500/all and platelet count ≥ 50,000/all. For second occurrence, alemtuzumab therapy should be withheld and resumed when ANC ≥ 500/all and platelet count ≥ 50,000/all at a dose of 10mg. Consider using reduce doses of MMF during alemtuzumab administration.

Basiliximab	Myelotoxicity is less as compared to rituximab, ATG and Alemtuzumab. Leucopenia occurs in approximately 10%–15% and thrombocytopenia 5% [76, 77].	It is the preferred agent in setting of hematological cytopenia. Reduction of MMF or anti-CMV medications may be considered in face of persisting leucopenia.

Daclizumab	Myelotoxicity is less and leukocyte, platelet and lymphocyte counts are significantly higher when compared with [78]	It is the preferred agent in setting of hematological cytopenia. Reduction of MMF or anti-CMV medications may be considered in face of persisting leucopenia.

Mycophenolate mofetil (MMF)	It causes leucopenia in 11.8% to 40% of KTR [79–82]. Other manifestation include anemia, thrombocytopenia and pancytopenia which and is frequent cause of dose reduction [23].	Consider reducing dose or holding MMF temporarily. Anti CMV medications dose reduction or holding it temporarily is also suggested. Reduce dose of MMF with concurrent use of ATG or alemtuzumab may prevent occurrence of hematological cytopenia.

Calcineurin inhibitors	Tacrolimus hematologic abnormalities occur in 16.92% and include anemia, neutropenia and combined neutropenia and thrombocytopenia [10]. Neutropenia is more (28%) in combination with MMF [19].Other manifestation of tacrolimus and cyclosporine include thrombotic microangiopathy[83–87]	Consider changing tacrolimus to cyclosporine in setting of persistent neutropenia. Reducing dose of MMF with tacrolimus may be considered due to pharmacodynamic and pharmacokinetic interaction between the two agents. In setting of thrombotic microangiopathy consider to change to MTORi or using belatacept. Eculizumab may be considered in setting of thrombotic microangiopathy.

Azathioprine	AZA causes leucopenia / neutropenia in around 50% of KTR [88].Frequency of leucopenia increases significantly following azathioprine dosage exceeding 1.99 mg/kg body weight/day [89].It can cause macrocytosis and megaloblastic changes in bone marrow which in turn can lead to ineffective erythropoiesis and pancytopenia [90–92]	Consider complete blood counts, including platelet counts, weekly during the first month, twice monthly for the second and third months of treatment, then monthly or more frequently if dosage alterations or other therapy changes are necessary. TPMT genotyping or phenotyping can help identify patients who are at an increased risk of developing AZA toxicity. Avoid using allopurinol with AZA. Reduce the dose of AZA if leucopenia persists.

Mammalian Target of rapamycin Inhibitors (MTOR inhibitors)	MTOR inhibitors causes post-transplant anemia [93] via impair metabolism and absorption [94, 95].They also causes leucopenia and thrombocytopenia [96]. The incidence of leucopenia and thrombocytopenia with everolimus is 11-19% and 10-17% respectively [97]. Both sirolimus [98–100] and everolimus [101, 102] causes thrombotic microangiopathy.	Consider reducing MMF dose and adjusting MTORi to lowest therapeutic level.

Valganciclovir	Neutropenia in 4.9% to 37.5% [3, 103–105] and leucopenia in 10-28% [3, 103, 106–108]. It also causes anemia and thrombocytopenia.	Consider using 450 mg once a day. Dose reduction or temporarily holding the medication can be considered in case of unresolving leucopenia.

Ganciclovir	The incidence of leucopenia is 7.1% to 23.1% [106, 109]. The incidence of thrombocytopenia and anemia is 23.1% and 38.5% [106]. Myelotoxicity is less as compared to valganciclovir [110].	Use correct doses according to graft function. Consider reducing dose. MMF dose reduction or holding it temporarily during ganciclovir treatment for CMV disease may be considered.

Valaciclovir	Bone marrow toxicity with valaciclovir is mild as compared to valganciclovir or ganciclovir. It causes anemia in 11-14% and leucopenia in 6-14% which was not significant from placebo [111].	Less myelotoxic and chances of cytopenia are not more than placebo.

Trimethoprim-Sulphamethaxazole (TMP-SMZ)	It causes blood cytopenia including neutropenia/leucopenia, thrombocytopenia and megaloblastic anemia. Trimethoprim inhibits granulopoiesis and erythropoiesis in vitro in a dose-dependent [112].It causes leucopenia in 39.61%, thrombocytopenia in 18.18% and neutropenia among leukopenic patients in 10.38% [13].	Consider alternative (atovaquone, dapsone and pentamidine) for *Pneumocystis jirovecii* prophylaxis.

Dapsone	It causes neutropenia [113, 114], agranulocytosis [115, 116] and methemoglobinemia[117–121].The incidence of dapsone induced methemoglobinemia in KTR is 46% [122].	Consider alternative (atovaquone, dapsone and pentamidine) for *Pneumocystis jirovecii* prophylaxis.

**Table 2:** Showing various studies done colony-stimulating factors.

Granulocyte Colony-Stimulating Factor (G-CSF)			
Author	Journal/Year	Study	Finding

Schmaldienst S et al.[244]	Transplantation / 2000	Author compared 30 episodes of leucopenia treated with G-CSF and compared them with age and sex matched historical control group in kidney transplant recipients.	Leukopenic episodes in treated groups were shorter, infections were significantly less and no evidence for triggering a rejection was found.

Peddi VR et al.[245]	Clin transplant / 1996	Retrospective analysis of 25 episodes of neutropenia in kidney or combined kidney and pancreas transplant who received G-CSF	Authors found G-CSF effective in reversing neutropenia and no evidence of rejection was found.

Turgeon N et al.[246]	Transpl Infect Dis/2000	Retrospective analysis of 50 patients (both kidney and liver transplant) who received 100 doses of G-CSF	It reversed neutropenia, allowed maximum doses of ganciclovir to treat CMV and was well tolerated. No relation was found between the highest WBC obtained during G-CSF therapy and the risk of rejection

Gordon MS et al.[250]	J Heart Lung Transplant / 1993	Febrile neutropenia in a heart transplant due to immunosuppressive medications	Neutrophil counts improved. Infection was successfully treated. Endomyocardial biopsy showed no rejection.

Ishizone S et al.[251]	J Pediatr Surg /1994	3 patients with severe liver disease and hypersplenism received G-CSF	G-CSF improved white cell counts without adverse events.

Foster PF et al.[252]	Transplantation / 1995	Prospective analysis of 37 primary liver allograft recipients received G-CSF for first 7 to 10 days.	Significant increase in white cell count, reduced rate of sepsis and sepsis related death were found in G-CSF group. The incidence of acute rejection was decreased in the G-CSF-treated group (22% vs. 51%, P < 0.01, chi-square test).

Winston DJ et al.[253]	Transplantation/1999	Randomized, placebo-controlled, double-blind, multicenter trial of efficacy and safety of granulocyte colony-stimulating factor in liver transplant recipients.	There was increase in white cell count. However there was no beneficial effect of G-CSF on infection, rejection and survival when compared to placebo.

Granulocyte Monocyte-Colony Stimulating Factor (GM-CSF)			

Hashmi A et al.[258]	Transplant Proc / 1997	7 patients with neutropenia were given GM-CSF and were compared with historical 7 control having neutropenia but have not received any GM-CSF	Mean leukocyte count was more in treated group. Infection, mean hospital stay and mortality was less in those who were treated with GM-CSF

Trindade E et al.[259]	Transplant Proc / 1997	13 children received 15 courses of GM-CSF	White cell count increased in all except one. No episode of rejection occurred. GM-CSF was found. It was beneficial in patients with severe bacterial infections.

Kutsogiannis DJ.[261]	Transplantation / 1992	Granulocyte macrophage colony-stimulating factor was used for the therapy of cytomegalovirus and ganciclovir-induced leucopenia in a renal transplant recipient.	It was helpful for improving white cell count and using adequate doses of anti CMV medications

Page AV. Et al.[262]	Curr Opin Organ Transplant /2008	Review article	Although there are encouraging results of G-CSF, GM-CSF and other immunomodulatory therapies in in vitro and in preclinical models still they have not met desired effects in solid organ transplantation and further studies are needed.

**Table 3:** Indications for platelet transfusion in thrombocytopenia.

Platelet count/Clinical scenario	Action / Platelet target

Thrombocytopenia with bleeding	Transfuse platelet

Platelet <10,000/ul	Transfuse [274–276]

Platelet count ≥ 10,000/ul and no bleeding	Observe [262, 274]

Thrombocytopenia for major surgery (Excluding neurosurgical procedure)	Keep platelet count Keep platelet ≥ 50,000/ul [277]

Gastroscopy and biopsy, insertion of indwelling lines, transbronchial biopsy, liver biopsy	Keep platelet ≥ 50,000/ul [278]

Thrombocytopenia planning for neurosurgery	Keep platelet ≥100,000 /ul [278]

Kidney Biopsy	Keep platelet ≥100,000 /ul [281]

Lumber puncture	Keep platelet ≥ 50,000/ul [280]

## Abbreviations

ANC: Absolute neutrophil count
ATG: Antithymocyte globulin (ATG)
AZA: Azathioprine
CTACE: Common terminology criteria for adverse events
EC-MPS: Enteric-coated mycophenolate sodium
G-CSF: Granulocyte colony stimulating factor
GM-CSF: Granulocyte-monocyte colony-stimulating factor
HHV-6: Herpesvirus-6
IL2-R antagonist: Interleukin receptor antagonist
KTR: Kidney transplant recipients
LON: Late-onset neutropenia
MTOR inhibitors: Mammalian target of rapamycin inhibitors
MMF: Mycophenolate mofetil
PVB19: Parvovirus B19
CMV: Cytomegalovirus
EBV: Epstein-Barr virus
TMP-SMZ: Trimethoprim-sulphamethaxazole.

## References

[1] Jafari A., Najivash P., Khatami M., Dashti-Khavidaki S. (2017). Cytopenia occurrence in kidney transplant recipients within early post-transplant period. *Journal of Research in Pharmacy Practice*.

[2] Yang Y., Yu B., Chen Y. (2015). Blood disorders typically associated with renal transplantation. *Frontiers in Cell and Developmental Biology*.

[3] Brum S., Nolasco F., Sousa J. (2008). Leukopenia in Kidney Transplant Patients With the Association of Valganciclovir and Mycophenolate Mofetil. *Transplantation Proceedings*.

[4] Wu S.-W., Chang H.-R., Lai Y.-R., Lian J.-D. (2008). Non-Life-Threatening Leukopenia in a Renal Transplant Recipient With Acute Overdose of Mycophenolate Mofetil. *Transplantation Proceedings*.

[5] Shin B. C., Chung J. H., Kim H. L. (2013). Sirolimus: A switch option for mycophenolate mofetil-induced leukopenia in renal transplant recipients. *Transplantation Proceedings*.

[6] Danziger-Isakov L., Baillie M. G. (2009). Hematologic complications of anti-CMV therapy in solid organ transplant recipients. *Clinical Transplantation*.

[7] Said T., Nampoory M. R. N., Pacsa A. S. (2007). Oral Valgancyclovir Versus Intravenous Gancyclovir for Cytomegalovirus Prophylaxis in Kidney Transplant Recipients. *Transplantation Proceedings*.

[8] Danesi R., Del Tacca M. (2004). Hematologic toxicity of immunosuppressive treatment. *Transplantation Proceedings*.

[9] Radivojević D., Blagojević-Lazić R., Ristić S., Laušević M., Ležaić V. (2011). Efficacy and safety of single and multiple dose antithymocyte globulin induction treatment in living related renal transplantation. *Biomedicine & Aging Pathology*.

[10] Dobrolet N. C., Webber S. A., Blatt J. (2001). Hematologic abnormalities in children and young adults receiving tacrolimus-based immunosuppression following cardiothoracic transplantation. *Pediatric Transplantation*.

[11] De Rycke A., Dierickx D., Kuypers D. R. (2011). Tacrolimus-induced neutropenia in renal transplant recipients. *Clinical Journal of the American Society of Nephrology*.

[12] Hong J. C., Kahan B. D. (2000). Sirolimus-induced thrombocytopenia and leukopenia in renal transplant recipients: Risk factors, incidence, progression, and management. *Transplantation*.

[13] Keisu M., Wiholm B.-E., Palmblad J. (1990). Trimethoprim-sulphamethoxazole-associated blood dyscrasias. Ten years' experience of the Swedish spontaneous reporting system. *Journal of Internal Medicine*.

[14] Gabardi S., Millen P., Hurwitz S., Martin S., Roberts K., Chandraker A. (2012). Atovaquone versus trimethoprim-sulfamethoxazole as Pneumocystis jirovecii pneumonia prophylaxis following renal transplantation. *Clinical Transplantation*.

[15] A Comprehensive listing. Available from: http://www.evs.nci.nih.gov/ftp1/CTCAE/CTCAE_4.03_20100614_QuickReference_8.5x11.pdf

[16] Dale D. C., Cottle T. E., Fier C. J. (2003). Severe chronic neutropenia: Treatment and follow-up of patients in the Severe Chronic Neutropenia International Registry. *American Journal of Hematology*.

[17] Bodey G. P., Buckley M., Sathe Y. S., Freireich E. J. (1966). Quantitative relationships between circulating leukocytes and infection in patients with acute leukemia.. *Annals of Internal Medicine*.

[18] Brown A. E. (1984). Neutropenia, fever, and infection. *American Journal of Medicine*.

[19] Zafrani L., Truffaut L., Kreis H. (2009). Incidence, risk factors and clinical consequences of neutropenia following kidney transplantation: A retrospective study. *American Journal of Transplantation*.

[20] Pelletier R. P., Akin B., Henry M. L. (2003). The impact of mycophenolate mofetil dosing patterns on clinical outcome after renal transplantation. *Clinical Transplantation*.

[21] De Souza R. M., Olsburgh J. (2008). Urinary tract infection in the renal transplant patient. *Nature Clinical Practice Nephrology*.

[22] Knoll G. A., Macdonald I., Khan A., Van Walraven C. (2003). Mycophenolate mofetil dose reduction and the risk of acute rejection after renal transplantation. *Journal of the American Society of Nephrology*.

[23] Vanhove T., Kuypers D., Claes K. J. (2013). Reasons for dose reduction of mycophenolate mofetil during the first year after renal transplantation and its impact on graft outcome. *Transplant International*.

[24] Taylor R. P., Lindorfer M. A. (2007). Drug Insight: The mechanism of action of rituximab in autoimmune disease - The immune complex decoy hypothesis. *Nature Clinical Practice Rheumatology*.

[25] Sonnenday C. J., Warren D. S., Cooper M. (2004). Plasmapheresis, CMV hyperimmune globulin, and anti-CD20 allow ABO-incompatible renal transplantation without splenectomy. *American Journal of Transplantation*.

[26] Shirakawa H., Ishida H., Shimizu T. (2011). The low dose of rituximab in ABO-incompatible kidney transplantation without a splenectomy: A single-center experience. *Clinical Transplantation*.

[27] Fuchinoue S., Ishii Y., Sawada T. (2011). The 5-year outcome of ABO-incompatible kidney transplantation with rituximab induction. *Transplantation*.

[28] Zarkhin V., Li L., Kambham N., Sigdel T., Salvatierra O., Sarwal M. M. (2008). A randomized, prospective trial of rituximab for acute rejection in pediatric renal transplantation. *American Journal of Transplantation*.

[29] Fehr T., Rüsi B., Fischer A., Hopfer H., Wüthrich R. P., Gaspert A. (2009). Rituximab and intravenous immunoglobulin treatment of chronic antibody-mediated kidney allograft rejection. *Transplantation*.

[30] Rostaing L., Guilbeau-Frugier C., Fort M., Mekhlati L., Kamar N. (2009). Treatment of symptomatic transplant glomerulopathy with rituximab. *Transplant International*.

[31] Svoboda J., Kotloff R., Tsai D. E. (2006). Management of patients with post-transplant lymphoproliferative disorder: The role of rituximab. *Transplant International*.

[32] https://www.gene.com/download/pdf/rituxan_prescribing.pdf, accessed on 19 April 2017

[33] Ram R., Bonstein L., Gafter-Gvili A., Ben-Bassat I., Shpilberg O., Raanani P. (2009). Rituximab-associated acute thrombocytopenia: an under-diagnosed phenomenon. *American Journal of Hematology*.

[34] Winkler U., Jensen M., Manzke O., Schulz H., Diehl V., Engert A. (1999). Cytokine-release syndrome in patients with B-cell chronic lymphocytic leukemia and high lymphocyte counts after treatment with an anti-CD20 monoclonal antibody (rituximab, IDEC-C2B8). *Blood*.

[35] Larrar S., Guitton C., Willems M., Bader-Meunier B. (2006). Severe hematological side effects following Rituximab therapy in children. *Haematologica*.

[36] Kimby E. (2005). Tolerability and safety of rituximab (MabThera). *Cancer Treatment Reviews*.

[37] Barnett A. N. R., Hadjianastassiou V. G., Mamode N. (2013). Rituximab in renal transplantation. *Transplant International*.

[38] Shah C., Grethlein S. J. (2004). Case Report of Rituximab-induced Thrombocytopenia. *American Journal of Hematology*.

[39] Otrock Z. K., Mahfouzd R. A. R., Oghlakian G. O., Salem Z. M., Bazarbachi A. (2005). Rituximab-induced acute thrombocytopenia: a report of two cases. *Haematologica*.

[40] Pamuk G. E., Donmez S., Turgut B., Demir M., Vural O. (2005). Rituximab-induced acute thrombocytopenia in a patient with prolymphocytic leukemia. *American Journal of Hematology*.

[41] Thachil J., Mukherje K., Woodcock B. (2006). Rituximab-induced haemorrhagic thrombocytopenia in a patient with hairy cell leukaemia. *British Journal of Haematology*.

[42] Van Den Hoogen M. W. F., Kamburova E. G., Baas M. C. (2015). Rituximab as induction therapy after renal transplantation: a randomized, double-blind, placebo-controlled study of efficacy and safety. *American Journal of Transplantation*.

[43] Ahmadi F., Dashti-Khavidaki S., Khatami M.-R., Lessan-Pezeshki M., Khalili H., Khosravi M. (2017). Rituximab-related late-onset neutropenia in kidney transplant recipients treated for antibody-mediated acute rejection. *Experimental and Clinical Transplantation*.

[44] Waiser J., Budde K., Schütz M. (2012). Comparison between bortezomib and rituximab in the treatment of antibody-mediated renal allograft rejection. *Nephrology Dialysis Transplantation *.

[45] Mitsuhata N., Fujita R., Ito S., Mannami M., Keimei K. (2005). Delayed-onset neutropenia in a patient receiving rituximab as treatment for refractory kidney transplantation. *Transplantation*.

[46] Dunleavy K., Hakim F., Kim H. K. (2005). B-cell recovery following rituximab-based therapy is associated with perturbations in stromal derived factor-1 and granulocyte homeostasis. *Blood*.

[47] Nitta E., Izutsu K., Sato T. (2007). A high incidence of late-onset neutropenia following rituximab-containing chemotherapy as a primary treatment of CD20-positive B-cell lymphoma: A single-institution study. *Annals of Oncology*.

[48] Lai G. G. Y., Lim S.-T., Tao M., Chan A., Li H., Quek R. (2009). Late-onset neutropenia following RCHOP chemotherapy in diffuse large B-cell lymphoma. *American Journal of Hematology*.

[49] Lemieux B., Tartas S., Traulle C. (2004). Rituximab-related late-onset neutropenia after autologous stem cell transplantation for aggressive non-Hodgkin's lymphoma. *Bone Marrow Transplantation*.

[50] Voog E., Morschhauser F., Solal-Céligny P., Benyunes M. C., Multani P. S., Saunders A. (2003). Neutropenia in patients treated with rituximab. *The New England Journal of Medicine*.

[51] Chaiwatanatorn K., Lee N., Grigg A., Filshie R., Firkin F. (2003). Delayed-onset neutropenia associated with rituximab therapy. *British Journal of Haematology*.

[52] Rios-Fernández R., Gutierrez-Salmerón M., Callejas-Rubio J., Fernández-Pugnaire M., Ortego-Centeno N. (2007). Late-onset neutropenia following rituximab treatment in patients with autoimmune diseases. *British Journal of Dermatology*.

[53] Cattaneo C., Spedini P., Casari S. (2006). Delayed-onset peripheral blood cytopenia after rituximab: Frequency and risk factor assessment in a consecutive series of 77 treatments. *Leukemia & Lymphoma*.

[54] Tesfa D., Palmblad J. (2011). Late-onset neutropenia following rituximab therapy: Incidence, clinical features and possible mechanisms. *Expert Review of Hematology*.

[55] Wolach O., Bairey O., Lahav M. (2010). Late-onset neutropenia after rituximab treatment: Case series and comprehensive review of the literature. *Medicine*.

[56] Ishida H., Inui M., Furusawa M., Tanabe K. (2013). Late-onset neutropenia (LON) after low-dose rituximab treatment in living related kidney transplantation - Single-center study. *Transplant Immunology*.

[57] Kabei K., Uchida J., Iwai T. (2014). Late-onset neutropenia and acute rejection in ABO-incompatible kidney transplant recipients receiving rituximab and mycophenolate mofetil. *Transplant Immunology*.

[58] Faguer S., Kamar N., Guilbeaud-Frugier C. (2007). Rituximab therapy for acute humoral rejection after kidney transplantation. *Transplantation*.

[59] Tanabe K. (2007). Japanese experience of ABO-incompatible living kidney transplantation. *Transplantation*.

[60] Kohei N., Hirai T., Omoto K., Ishida H., Tanabe K. (2012). Chronic antibody-mediated rejection is reduced by targeting B-cell immunity during an introductory period. *American Journal of Transplantation*.

[61] Cheungpasitporn W., Thongprayoon C., Edmonds P. J., Bruminhent J., Tangdhanakanond K. (2015). The effectiveness and safety of rituximab as induction therapy in ABO-compatible non-sensitized renal transplantation: A systematic review and meta-analysis of randomized controlled trials. *Renal Failure*.

[62] Dhand S., Bahrain H. (2008). Rituximab-induced severe acute thrombocytopenia: A case report and review of literature. *Cancer Investigation*.

[63] Provan D., Stasi R., Newland A. C. (2010). International consensus report on the investigation and management of primary immune thrombocytopenia. *Blood*.

[64] Lebranchu Y., Bridoux F., Büchler M. (2002). Immunoprophylaxis with basiliximab compared with antithymocyte globulin in renal transplant patients receiving MMF-containing triple therapy. *American Journal of Transplantation*.

[65] Gurk-Turner C., Airee R., Philosophe B., Kukuruga D., Drachenberg C., Haririan A. (2008). Thymoglobulin dose optimization for induction therapy in high risk kidney transplant recipients. *Transplantation*.

[66] Brennan D. C., Daller J. A., Lake K. D., Cibrik D., Del Castillo D. (2006). Rabbit antithymocyte globulin versus basiliximab in renal transplantation. *The New England Journal of Medicine*.

[67] Woodle E. S., Moore L. W. (1998). Results of the double blinded , randomized, multicenter phase III clinical trial of thymoglobulin versus ATGAM in the treatment of acute graft rejection episodes after renal transplantation. *Transplantation*.

[68] Beiras-Fernandez A., Thein E., Chappel D. (2004). Polyclonal anti-thymocyte globulins influence apoptosis in reperfused tissues after ischaemia in a non-human primate model. *Transplant International*.

[69] http://products.sanofi.ca/en/thymoglobulin.pdf., Accessed on 25/04/17

[70] Shamsaeefar A., Roozbeh J., Khajerezae S. (2016). Effects of induction therapy with alemtuzumab versus antithymocyte globulin among highly sensitized kidney transplant candidates. *Saudi journal of kidney diseases and transplantation : an official publication of the Saudi Center for Organ Transplantation, Saudi Arabia*.

[71] Hartmann E. L., Gatesman M., Roskopf-Somerville J., Stratta R., Farney A., Sundberg A. (2008). Management of leukopenia in kidney and pancreas transplant recipients. *Clinical Transplantation*.

[72] Smith A., Couvillion R., Zhang R. (2014). Incidence and Management of Leukopenia/Neutropenia in 233 Kidney Transplant Patients Following Single Dose Alemtuzumab Induction. *Transplantation Proceedings*.

[73] Muthusamy A. S. R., Vaidya A. C., Sinha S., Roy D., Elker D. E., Friend P. J. (2008). Alemtuzumab induction and steroid-free maintenance immunosuppression in pancreas transplantation. *American Journal of Transplantation*.

[74] Havrdova E., Horakova D., Kovarova I. (2015). Alemtuzumab in the treatment of multiple sclerosis: Key clinical trial results and considerations for use. *Therapeutic Advances in Neurological Disorders*.

[75] Haider I., Cahill M. (2004). Fatal thrombocytopenia temporally related to the administration of alemtuzumab (MabCampath) for refractory CLL despite early discontinuation of therapy. *International Journal of Hematology*.

[76] Zaza G., Tomei P., Granata S., Boschiero L., Lupo A. (2014). Monoclonal antibody therapy and renal transplantation: Focus on adverse effects. *Toxins*.

[77] Chen G., Gu J., Qiu J. (2013). Efficacy and safety of thymoglobulin and basiliximab in kidney transplant patients at high risk for acute rejection and delayed graft function. *Experimental and Clinical Transplantation*.

[78] Noël C., Abramowicz D., Durand D. (2009). Daclizumab versus antithymocyte globulin in high-immunological-risk renal transplant recipients. *Journal of the American Society of Nephrology*.

[79] Kuypers D. R. J., Claes K., Evenepoel P., Maes B., Vanrenterghem Y. (2004). Clinical efficacy and toxicity profile of tacrolimus and mycophenolic acid in relation to combined long-term pharmacokinetics in de novo renal allograft recipients. *Clinical Pharmacology & Therapeutics*.

[80] Le Meur Y., Büchler M., Thierry A. (2007). Individualized mycophenolate mofetil dosing based on drug exposure significantly improves patient outcomes after renal transplantation. *American Journal of Transplantation*.

[81] van Gelder T., Hilbrands L. B., Vanrenterghem Y. (1999). A randomized double-blind, multicenter plasma concentration controlled study of the safety and efficacy of oral mycophenolate mofetil for the prevention of acute rejection after kidney transplantation. *Transplantation*.

[82] Gaston R. S., Kaplan B., Shah T. (2009). Fixed- or controlled-dose mycophenolate mofetil with standard- or reduced-dose calcineurin inhibitors: The opticept trial. *American Journal of Transplantation*.

[83] Pham P.-T. T., Peng A., Wilkinson A. H. (2000). Cyclosporine and tacrolimus-associated thrombotic microangiopathy. *American Journal of Kidney Diseases*.

[84] Cortina G., Trojer R., Waldegger S., Schneeberger S., Gut N., Hofer J. (2015). De novo tacrolimus-induced thrombotic microangiopathy in the early stage after renal transplantation successfully treated with conversion to everolimus. *Pediatric Nephrology*.

[85] Carson J. M., Newman E. D., Farber J. L., Filippone E. J. (2012). Tacrolimus-induced thrombotic microangiopathy: Natural history of a severe, acute vasculopathy. *Clinical Nephrology*.

[86] Merola J., Yoo P. S., Schaub J. (2016). Belatacept and Eculizumab for Treatment of Calcineurin Inhibitor-induced Thrombotic Microangiopathy After Kidney Transplantation: Case Report. *Transplantation Proceedings*.

[87] Commereuc M., Karras A., Amrein C. (2013). Successful treatment of acute thrombotic microangiopathy by eculizumab after combined lung and kidney transplantation. *Transplantation*.

[88] Pollak R., Nishikawa R. A., Mozes M. F., Jonasson O. (1980). Azathioprine-induced leukopenia—Clinical significance in renal transplantation. *Journal of Surgical Research*.

[89] Oesterwitz H., Horpacsy G., May G., Mebel M. (1978). Frequency of leukopenia incidents following azathioprine therapy after kidney transplantation. *European Urology*.

[90] Kim C. J., Park K. I., Inoue H. (1998). Azathioprine-induced megaloblastic anemia with pancytopenia 22 years after living-related renal transplantation. *International Journal of Urology*.

[91] Wlckramasinghe S. N., Dodsworth H., Rault R. M. J., Hulme B. (1974). Observations on the incidence and cause of macrocytosis in patients on azathioprine therapy following renal transplantation. *Transplantation*.

[92] Sjogren U., Thysell H., Lindholm T. (1981). Bone Marrow Morphology in Patients in Long‐Term Treatment with Azathioprine. *European Journal of Haematology*.

[93] Sánchez Fructuoso A., Calvo N., Moreno M. A., Giorgi M., Barrientos A. (2007). Study of Anemia After Late Introduction of Everolimus in the Immunosuppressive Treatment of Renal Transplant Patients. *Transplantation Proceedings*.

[94] Sofroniadou S., Kassimatis T., Goldsmith D. (2010). Anaemia, microcytosis and sirolimus—is iron the missing link?. *Nephrology Dialysis Transplantation*.

[95] Fishbane S., Cohen D. J., Coyne D. W., Djamali A., Singh A. K., Wish J. B. (2009). Posttransplant anemia: The role of sirolimus. *Kidney International*.

[96] Webster A. C., Lee V. W. S., Chapman J. R., Craig J. C. (2006). Target of rapamycin inhibitors (sirolimus and everolimus) for primary immunosuppression of kidney transplant recipients: A systematic review and meta-analysis of randomized trials. *Transplantation*.

[97] Kovarik J. M., Kaplan B., Silva H. T. (2002). Exposure-response relationships for everolimus in de novo kidney transplantation: Defining a therapeutic range. *Transplantation*.

[98] Yilmaz V. T., Koçak H., Avci A. B., Salim O., Ersoy F. F., Süleymanlar G. (2011). Thrombotic thrombocytopenic purpura associated with everolimus use in a renal transplant patient. *International Urology and Nephrology*.

[99] Lovric S., Kielstein J. T., Kayser D. (2011). Combination of everolimus with calcineurin inhibitor medication resulted in post-transplant haemolytic uraemic syndrome in lung transplant recipients—a case series. *Nephrology Dialysis Transplantation*.

[100] Reese J. A., Bougie D. W., Curtis B. R. (2015). Drug-induced thrombotic microangiopathy: Experience of the Oklahoma registry and the BloodCenter of Wisconsin. *American Journal of Hematology*.

[101] Barone G. W., Gurley B. J., Abul-Ezz S. R., Gökden N. (2003). Sirolimus-induced thrombotic microangiopathy in a renal transplant recipient. *American Journal of Kidney Diseases*.

[102] Sartelet H., Toupance O., Lorenzato M. (2005). Sirolimus-induced thrombotic microangiopathy is associated with decreased expression of vascular endothelial growth factor in kidneys. *American Journal of Transplantation*.

[103] Gabardi S., Magee C. C., Baroletti S. A., Powelson J. A., Cina J. L., Chandraker A. K. (2004). Efficacy and safety of low-dose valganciclovir for prevention of cytomegalovirus disease in renal transplant recipients: A single-center, retrospective analysis. *Pharmacotherapy*.

[104] Rerolle J.-P., Szelag J.-C., Le Meur Y. (2007). Unexpected rate of severe leucopenia with the association of mycophenolate mofetil and valganciclovir in kidney transplant recipients. *Nephrology Dialysis Transplantation*.

[105] Chen I.-M., Chang H.-H., Hsu C.-P., Lai S.-T., Hsieh Y.-C., Shih C.-C. (2007). Correlation between body mass index and leucopenia after administration of valganciclovir for cytomegalovirus infection in Chinese cardiac recipients. *Circulation Journal*.

[106] Paya C., Humar A., Dominguez E. (2004). Efficacy and Safety of Valganciclovir vs. Oral Ganciclovir for Prevention of Cytomegalovirus Disease in Solid Organ Transplant Recipients. *American Journal of Transplantation*.

[107] Pescovitz M. D., Rabkin J., Merion R. M. (2000). Valganciclovir Results in Improved Oral Absorption of Ganciclovir in Liver Transplant Recipients. *Antimicrobial Agents and Chemotherapy*.

[108] Humar A., Lebranchu Y., Vincenti F. (2010). The efficacy and safety of 200 days valganciclovir cytomegalovirus prophylaxis in high-risk kidney transplant recipients. *American Journal of Transplantation*.

[109] Reischig T., Opatrny K., Bouda M., Treska V., Jindra P., Svecova M. (2002). A randomized prospective controlled trial of oral ganciclovir versus oral valacyclovir for prophylaxis of cytomegalovirus disease after renal transplantation. *Transplant International*.

[110] Kalil A. C., Freifeld A. G., Lyden E. R., Stoner J. A. (2009). Valganciclovir for cytomegalovirus prevention in solid organ transplant patients: An evidence-based reassessment of safety and efficacy. *PLoS ONE*.

[111] Lowance D., Neumayer H.-H., Legendre C. M. (1999). Valacyclovir for the prevention of cytomegalovirus disease after renal transplantation. International Valacyclovir Cytomegalovirus Prophylaxis Transplantation Study Group. *The New England Journal of Medicine*.

[112] Golde D. W., Bersch N., Quan S. G. (1978). Trimethoprim and Sulphamethoxazole Inhibition of Haematopoiesis in Vitro. *British Journal of Haematology*.

[113] Raizman M. B., Fay A. M., Weiss J. S. (1994). Dapsone-induced Neutropenia in Patients Treated for Ocular Cicatricial Pemphigoid. *Ophthalmology*.

[114] Sabnis G. R., Kulkarni U. P., Gokhale Y. A. (2012). Dapsone-induced neutropenia with invasive pulmonary aspergillosis. *Lung India*.

[115] Coleman M. D. (2001). Dapsone-mediated agranulocytosis: Risks, possible mechanisms and prevention. *Toxicology*.

[116] Kobe Y., Setoguchi D., Kitamura N. (2011). Dapsone-induced agranulocytosis leading to perianal abscess and death: A case report. *Journal of Medical Case Reports*.

[117] Ganer A., Knobel B., Fryd C. H., Rachmilewitz E. A. (1981). Dapsone-induced methemoglobinemia and hemolysis in the presence of familial hemoglobinopathy Hasharon and familial methemoglobin reductase deficiency. *Israel Journal of Medical Sciences *.

[118] Burke P., Jahangir K., Kolber M. R. (2013). Dapsone-induced methemoglobinemia: Case of the blue lady. *Canadian Family Physician*.

[119] Singh S., Sethi N., Pandith S., Ramesh G. S. (2014). Dapsone-induced methemoglobinemia: "saturation gap"-The key to diagnosis. *Journal of Anaesthesiology Clinical Pharmacology*.

[120] Ashurst J. V., Wasson M. N., Hauger W., Fritz W. T. (2010). Pathophysiologic mechanisms, diagnosis, and management of dapsone-induced methemoglobinemia. *The Journal of the American Osteopathic Association*.

[121] Ward K. E., McCarthy M. W. (1998). Dapsone-induced methemoglobinemia. *Annals of Pharmacotherapy*.

[122] Mitsides N., Green D., Middleton R. (2014). Dapsone-induced methemoglobinemia in renal transplant recipients: More prevalent than previously thought. *Transplant Infectious Disease*.

[123] Préville X., Flacher M., LeMauff B. (2001). Mechanisms involved in antithymocyte globulin immunosuppressive activity in a nonhuman primate model. *Transplantation*.

[124] Préville X., Nicolas L., Flacher M., Revillard J.-P. (2000). A quantitative flow cytometry assay for the preclinical testing and pharmacological monitoring of rabbit antilymphocyte globulins (rATG). *Journal of Immunological Methods*.

[125] Agha I. A., Rueda J., Alvarez A. (2002). Short course induction immunosuppression with thymoglobulin for renal transplant recipients. *Transplantation*.

[126] Swanson S. J., Hale D. A., Mannon R. B. (2002). Kidney transplantation with rabbit antithymocyte globulin induction and sirolimus monotherapy. *The Lancet*.

[127] Starzl T. E., Murase N., Abu-Elmagd K. (2003). Tolerogenic immunosuppression for organ transplantation. *The Lancet*.

[128] Ata P., Kara M., Özdemir E. (2013). Monitoring of CD3+ T-cell count in patients receiving antithymocyte globulin induction after cadaveric renal transplantation. *Transplantation Proceedings*.

[129] Gorrie M., Thomson G. (1997). Dose titration during anti-thymocyte globulin therapy: Monitoring by CD3 count or total lymphocyte count?. *Clinical Laboratory Haematology*.

[130] Clark K. R., Forsythe J. L. R., Shenton B. K., Lennard T. W. J., Proud G., Taylor R. M. R. (1993). Administration of ATG according to the absolute T lymphocyte count during therapy for steroid-resistant rejection. *Transplant International*.

[131] Krasinskas A. M., Kreisel D., Acker M. A. (2002). CD3 monitoring of antithymocyte globulin therapy in thoracic organ transplantation. *Transplantation*.

[132] Cantarovich D., Giral-Classe M., Hourmant M. (2000). Prevention of acute rejection with antithymocyte globulin, avoiding corticosteriods, and delaying cyclosporin after renal transplantation. *Nephrology Dialysis Transplantation *.

[133] Glotz D., Charpentier B., Abramovicz D. (2010). Thymoglobulin induction and sirolimus versus tacrolimus in kidney transplant recipients receiving mycophenolate mofetil and steroids. *Transplantation*.

[134] Woodle E. S., Peddi V. R., Tomlanovich S., Mulgaonkar S., Kuo P. C. (2010). A prospective, randomized, multicenter study evaluating early corticosteroid withdrawal with Thymoglobulin® in living-donor kidney transplantation. *Clinical Transplantation*.

[135] Matl I., Lácha J., Lodererová A. (2000). Withdrawal of steroids from triple-drug therapy in kidney transplant patients. *Nephrology Dialysis Transplantation *.

[136] Flynn J. M., Byrd J. C. (2000). Alemtuzumab monoclonal antibody therapy. *Current Opinion in Oncology*.

[137] Ratzinger G., Reagan J. L., Heller G., Busam K. J., Young J. W. (2003). Differential CD52 expression by distinct myeloid dendritic cell subsets: implications for alemtuzumab activity at the level of antigen presentation in allogeneic graft-host interactions in transplantation. *Blood*.

[138] Calne R., Friend P., Moffatt S. (1998). Prope tolerance, perioperative campath 1H, and low-dose cyclosporin monotherapy in renal allograft recipients. *The Lancet*.

[139] Tan H. P., Donaldson J., Basu A. (2009). Two hundred living donor kidney transplantations under alemtuzumab induction and tacrolimus monotherapy: 3-Year follow-up. *American Journal of Transplantation*.

[140] Watson C. J. E., Bradley J. A., Friend P. J. (2005). Alemtuzumab (CAMPATH 1H) induction therapy in cadaveric kidney transplantation—efficacy and safety at five years. *American Journal of Transplantation*.

[141] Hanaway M. J., Woodle E. S., Mulgaonkar S. (2011). Alemtuzumab induction in renal transplantation. *The New England Journal of Medicine*.

[142] Reams B. D., Musselwhite L. W., Zaas D. W. (2007). Alemtuzumab in the treatment of refractory acute rejection and bronchiolitis obliterans syndrome after human lung transplantation. *American Journal of Transplantation*.

[143] Ciancio G., Burke G. W., Gaynor J. J. (2005). A randomized trial of three renal transplant induction antibodies: Early comparison of tacrolimus, mycophenolate mofetil, and steroid dosing, and newer immune-monitoring. *Transplantation*.

[144] Bank J. R., Heidt S., Moes D. J. (2017). Alemtuzumab Induction and Delayed Acute Rejection in Steroid-Free Simultaneous Pancreas-Kidney Transplant Recipients. *Transplantation Direct*.

[145] http://www.campath.com/pdfs/2009-08-Campath%20US%20PI.pdf, Accessed on 29/04/17

[146] Sageshima J., Ciancio G., Chen L., Burke G. W. (2009). Anti-interleukin-2 receptor antibodies-basiliximab and daclizumab-for the prevention of acute rejection in renal transplantation. *Biologics : Targets & Therapy*.

[147] 3C Study Collaborative Group, Haynes R., Harden P. (2014). Alemtuzumab-based induction treatment versus basiliximab-based induction treatment in kidney transplantation (the 3C Study): a randomised trial. *The Lancet*.

[148] Kesiraju S., Paritala P., Rao Ch U. M., Athmakuri S. M., Reddy V. S., Sahariah S. (2014). Anti-thymocyte globulin versus basiliximab induction in renal transplant recipients: Long-term outcome.. *Saudi journal of kidney diseases and transplantation : an official publication of the Saudi Center for Organ Transplantation, Saudi Arabia*.

[149] Brennan D. C., Daller J. A., Lake K. D., Cibrik D., Del Castillo D. (2006). Thymoglobulin Induction Study Group. Rabbit antithymocyte globulin versus basiliximab in renal transplantation. *New England Journal of Medicine*.

[150] Carr S. F., Papp E., Wu J. C., Natsumeda Y. (1993). Characterization of human type I and type II IMP dehydrogenases. *The Journal of Biological Chemistry*.

[151] Allison A. C., Eugui E. M. (2000). Mycophenolate mofetil and its mechanisms of action. *International Journal of immunopharmacology*.

[152] European Mycophenolate Mofetil Cooperative Study Group (1995). Placebo-controlled study of mycophenolate mofetil combined with cyclosporin and corticosteroids for prevention of acute rejection. *The Lancet*.

[153] The Tricontinental Mycophenolate Mofetil Renal Transplantation Study Group (1996). A blinded, randomized clinical trial of mycophenolate mofetil for the prevention of acute rejection in cadaveric renal transplantation. *Transplantation*.

[154] Mudge D. W., Atcheson B. A., Taylor P. J., Pillans P. I., Johnson D. W. (2004). Severe toxicity associated with a markedly elevated mycophenolic acid free fraction in a renal transplant recipient. *Therapeutic Drug Monitoring*.

[155] Royer B., Zanetta G., Bérard M. (2003). A neutropenia suggesting an interaction between valacyclovir and mycophenolate mofetil. *Clinical Transplantation*.

[156] Alvarez P. A., Egozcue J., Sleiman J., Moretti L., Di Girolamo G., Keller G. A. (2012). Severe neutropenia in a renal transplant patient suggesting an interaction between mycophenolate and fenofibrate. *Current Drug Safety*.

[157] Jacobson P. A., Schladt D., Oettin W. S. (2011). Genetic determinants of mycophenolate-related anemia and leucopenia after transplantation. *Transplantation*.

[158] Matsui K., Shibagaki Y., Sasaki H., Chikaraishi T., Yasuda T., Kimura K. (2010). Mycophenolate mofetil-induced agranulocytosis in a renal transplant recipient. *Clinical and Experimental Nephrology*.

[159] Tierce J. C., Porterfield-Baxa J., Petrilla A. A., Kilburg A., Ferguson R. M. (2005). Impact of mycophenolate mofetil (MMF)-related gastrointestinal complications and MMF dose alterations on transplant outcomes and healthcare costs in renal transplant recipients. *Clinical Transplantation*.

[160] Kalil A. C., Mindru C., Florescu D. F. (2011). Effectiveness of valganciclovir 900 mg versus 450 mg for cytomegalovirus prophylaxis in transplantation: Direct and indirect treatment comparison meta-analysis. *Clinical Infectious Diseases*.

[161] Savvidaki E., Kazakopoulos P., Papachristou E. (2014). Replacement of mycophenolate acid with everolimus in patients who became neutropenic after renal transplant. *Experimental and Clinical Transplantation*.

[162] Webster A. C., Woodroffe R. C., Taylor R. S., Chapman J. R., Craig J. C. (2005). Tacrolimus versus ciclosporin as primary immunosuppression for kidney transplant recipients: Meta-analysis and meta-regression of randomised trial data. *British Medical Journal*.

[163] Nosari A., Marbello L., De Carlis L. G. (2004). Bone Marrow Hypoplasia Complicating Tacrolimus (FK506) Therapy. *International Journal of Hematology*.

[164] Hirao A., Kawano Y., Takaue Y. (1993). Effects of immunosuppressants, FK506, deoxyspergualin, and cyclosporine A on immature human hematopoiesis. *Blood*.

[165] Koenig J. M., Matharoo N., Stegner J. J., Schowengerdt K. O. (2005). Tacrolimus: In vitro effects on myelopoiesis, apoptosis, and CD11b expression. *The Journal of Heart and Lung Transplantation*.

[166] Zucker K., Tsaroucha A., Olson L., Esquenazi V., Tzakis A., Miller J. (1999). Evidence that tacrolimus augments the bioavailability of mycophenolate mofetil through the inhibition of mycophenolic acid glucuronidation. *Therapeutic Drug Monitoring*.

[167] Balogh A., Merkel U., Müller D. (2003). Can xipamide or tacrolimus inhibit the glucuronidation of mycophenolic acid in rat liver slices?. *Experimental and Toxicologic Pathology*.

[168] Van Gelder T., Klupp J., Barten M. J., Christians U., Morris R. E. (2001). Comparison of the effects of tacrolimus and cyclosporine on the pharmacokinetics of mycophenolic acid. *Therapeutic Drug Monitoring*.

[169] Kuypers D. R. J., Vanrenterghem Y., Squifflet J. P. (2003). Twelve-month evaluation of the clinical pharmacokinetics of total and free mycophenolic acid and its glucuronide metabolites in renal allograft recipients on low dose tacrolimus in combination with mycophenolate mofetil. *Therapeutic Drug Monitoring*.

[170] Undre N. A., Van Hooff J., Christiaans M. (1998). Pharmacokinetics of FK 506 and mycophenolic acid after the administration of a FK 506-based regimen in combination with mycophenolate mofetil in kidney transplantation. *Transplantation Proceedings*.

[171] Yau W.-P., Vathsala A., Lou H.-X., Chan E. (2007). Is a standard fixed dose of mycophenolate mofetil ideal for all patients?. *Nephrology Dialysis Transplantation *.

[172] Suhail S. M., Vathsala A., Lou H. X., Woo K. T. (2000). Safety and efficacy of mycophenolate mofetil for prophylaxis in Asian renal transplant recipients. *Transplantation Proceedings*.

[173] Naito T. (2010). Optimal immunosuppressive therapy based on pharmacokinetics and pharmacodynamics of antimetabolites in clinical practice. *Yakugaku Zasshi*.

[174] Murray J. E., Merrill J. P., Dammin G. J., Dealy J. B., Alexandre G. W., Harrison J. H. (1962). Kidney transplantation in modified recipients. *Annals of Surgery*.

[175] Halloran P., Mathew T., Tomlanovich S., Groth C., Hooftman L., Barker C. (1997). Mycophenolate mofetil in renal allograft recipients: A pooled efficacy analysis of three randomized, double-blind, clinical studies in prevention of rejection. The International Mycophenolate Mofetil Renal Transplant Study Groups. *Transplantation*.

[176] Lennard L. (1992). The clinical pharmacology of 6-mercaptopurine. *European Journal of Clinical Pharmacology*.

[177] Weinshilboum R. (2001). Thiopurine pharmacogenetics: Clinical and molecular studies of thiopurine methyltransferase. *Drug Metabolism and Disposition*.

[178] McLeod H. L., Siva C. (2002). The thiopurine S-methyltansferase gene locus - Implications for clinical pharmacogenomics. *Pharmacogenomics*.

[179] Lennard L., Brown C., Fox M., Maddocks J. (1984). Azathioprine metabolism in kidney transplant recipients.. *British Journal of Clinical Pharmacology*.

[180] Anstey A., Lennard L., Mayou S. C., Kirby J. D. (1992). Pancytopenia related to azathioprine - An enzyme deficiency caused by a common genetic polymorphism: A review. *Journal of the Royal Society of Medicine*.

[181] Stolk J. N., Beorbooms A. M., de Abreu R. A. (1998). Reduced thiopurine methyltransferase activity and development of side effects of azathioprine treatment in patients with rheumatoid arthritis. *Arthritis & Rheumatology*.

[182] Yates C. R., Krynetski E. Y., Loennechen T. (1997). Molecular diagnosis of thiopurine S-methyltransferase deficiency: Genetic basis for azathioprine and mercaptopurine intolerance. *Annals of Internal Medicine*.

[183] Black A. J., McLeod H. L., Capell H. A. (1998). Thiopurine methyltransferase genotype predicts therapy-limiting severe toxicity from azathioprine. *Annals of Internal Medicine*.

[184] Clunie G. P. R., Lennard L. (2004). Relevance of thiopurine methyltransferase status in rheumatology patients receiving azathioprine. *Rheumatology*.

[185] Chocair P. R., Duley J. A., Simmonds H. A., Cameron J. S. (1992). The importance of thiopurine methyltransferase activity for the use of azathioprine in transplant recipients. *Transplantation*.

[186] Budhiraja P., Popovtzer M. (2011). Azathioprine-related myelosuppression in a patient homozygous for TPMT∗3A. *Nature Reviews Nephrology*.

[187] Kurzawski M., Dziewanowski K., Gawrońska-Szklarz B., Domański L., Droździk M. (2005). The impact of thiopurine S-methyltransferase polymorphism on azathioprine-induced myelotoxicity in renal transplant recipients. *Therapeutic Drug Monitoring*.

[188] Clive D. M. (2000). Renal transplant-associated hyperuricemia and gout. *Journal of the American Society of Nephrology*.

[189] Raman G. V., Sharman V. L., Lee H. A. (1990). Azathioprine and allopurinol: a potentially dangerous combination. *Journal of Internal Medicine*.

[190] http://www.kdigo.org/clinical_practice_guidelines/pdf/KITxpGL_summary.pdf, accessed on 14/05/2017

[191] https://www.accessdata.fda.gov/drugsatfda_docs/label/2011/016324s034s035lbl.pdf, accessed on 14/05/2017

[192] Augustine J. J., Knauss T. C., Schulak J. A., Bodziak K. A., Siegel C., Hricik D. E. (2004). Comparative effects of sirolimus and mycophenolate mofetil on erythropoiesis in kidney transplant patients. *American Journal of Transplantation*.

[193] Sofroniadou S., Goldsmith D. (2011). Mammalian target of rapamycin (mTOR) inhibitors: potential uses and a review of haematological adverse effects. *Drug Safety*.

[194] Liu J., Liu D., Li J. (2017). Efficacy and safety of everolimus for maintenance immunosuppression of kidney transplantation: A meta-analysis of randomized controlled trials. *PLoS ONE*.

[195] McDonald M. A., Gustafsson F., Almasood A., Barth D., Ross H. J. (2010). Sirolimus is associated with impaired hematopoiesis in heart transplant patients? A retrospective analysis. *Transplantation Proceedings*.

[196] Fernández Fresnedo G., Palomar R., Rodrigo E. (2005). Prevalence of anemia in renal transplant patients: Results from MOST, an observational trial. *Transplantation Proceedings*.

[197] Diekmann F., Rovira J., Diaz-Ricart M. (2012). MTOR inhibition and erythropoiesis: Microcytosis or anaemia?. *Nephrology Dialysis Transplantation *.

[198] Rostaing L., Kamar N. (2010). mTOR inhibitor/proliferation signal inhibitors: entering or leaving the field?. *Journal of Nephrology*.

[199] Vitko S., Tedesco H., Eris J. (2004). Everolimus with Optimized Cyclosporine Dosing in Renal Transplant Recipients: 6-Month Safety and Efficacy Results of Two Randomized Studies. *American Journal of Transplantation*.

[200] Dansirikul C., Duffull S. B., Morris R. G., Tett S. E. (2005). Relationships between sirolimus dosing, concentration and outcomes in renal transplant recipients. *British Journal of Clinical Pharmacology*.

[201] Groth C. G., Bäckman L., Morales J.-M. (1999). Sirolimus (rapamycin)-based therapy in human renal transplantation: similar efficacy and different toxicity compared with cyclosporine. Sirolimus European Renal Transplant Study Group. *Transplantation*.

[202] Jacobson P. A., Schladt D., Oetting W. S. (2011). Genetic Determinants of Mycophenolate-Related Anemia and Leukopenia After Transplantation. *Transplantation*.

[203] Kreis H., Cisterne J.-M., Land W. (2000). Sirolimus in association with mycophenolate mofetil induction for the prevention of acute graft rejection in renal allograft recipients. *Transplantation*.

[204] Vitko S., Wlodarczyk Z., Kyllönen L. (2006). Tacrolimus combined with two different dosages of sirolimus in kidney transplantation: Results of a multicenter study. *American Journal of Transplantation*.

[205] Grinyó J. M., Cruzado J. M. (2006). Mycophenolate mofetil and sirolimus combination in renal transplantation. *American Journal of Transplantation*.

[206] Flechner S. M., Friend P. J., Brockmann J. (2005). Alemtuzumab induction and sirolimus plus mycophenolate mofetil maintenance for CNI and steroid-free kidney transplant immunosuppression. *American Journal of Transplantation*.

[207] Holdaas H., Midtvedt K., Åsberg A. (2012). A drug safety evaluation of everolimus in kidney transplantation. *Expert Opinion on Drug Safety*.

[208] Paul S. R., Bennett F., Calvetti J. A. (1990). Molecular cloning of a cDNA encoding interleukin 11, a stromal cell-derived lymphopoietic and hematopoietic cytokine. *Proceedings of the National Acadamy of Sciences of the United States of America*.

[209] Quesniaux V. F. J., Wehrli S., Steiner C. (1994). The immunosuppressant rapamycin blocks in vitro responses to hematopoietic cytokines and inhibits recovering but not steady-state hematopoiesis in vivo. *Blood*.

[210] Waduud M. A., McMillan M. (2014). Valganciclovir-induced lecuopenia in renal transplant recipients treated with mycophenolate mofetil. *International Journal of Surgery*.

[211] Doyle A. M., Warburton K. M., Goral S., Blumberg E., Grossman R. A., Bloom R. D. (2006). 24-Week oral ganciclovir prophylaxis in kidney recipients is associated with reduced symptomatic cytomegalovirus disease compared to a 12-week course. *Transplantation*.

[212] Reischig T., Kacer M. (2014). The efficacy and cost-effectiveness of valacyclovir in cytomegalovirus prevention in solid organ transplantation. *Expert Review of Pharmacoeconomics & Outcomes Research*.

[213] Bjornson B. H., McLntyre A. P., Harvey J. M., Tauber A. I. (1986). Studies of the effects of trimethoprim and sulfamethoxazole on human granulopoiesis. *American Journal of Hematology*.

[214] Chanarin I., England J. M. (1972). Toxicity of Trimethoprim-Sulphamethoxazole in Patients with Megaloblastic Haemovoiesis. *British Medical Journal*.

[215] Jewkes R. F., Edwards M. S., Grant B. J. B. (1970). Haematological changes in a patient on long-term treatment with a trimethoprim-sulphonamide combination. *Postgraduate Medical Journal*.

[216] Principi N., Marchisio P., Biasini A., Villa Dalla A., Biasini G. (1984). Early and late neutropenia in children treated with cotrimoxazole (trimethoprim-sulfamethoxazole). *Acta Paediatrica Scandinavica*.

[217] Asmar B. I., Maqbool S., Dajani A. S. (1981). Hematologic Abnormalities After Oral Trimethoprim-Sulfamethoxazole Therapy in Children. *American Journal of Diseases of Children*.

[218] Fox B. C., Sollinger H. W., Belzer F. O., Maki D. G. (1990). A prospective, randomized, double-blind study of trimethoprim-sulfamethoxazole for prophylaxis of infection in renal transplantation: Clinical efficacy, absorption of trimethoprim-sulfamethoxazole, effects on the microflora, and the cost-benefit of prophylaxis. *American Journal of Medicine*.

[219] Mitsides N., Greenan K., Green D. (2014). Complications and outcomes of trimethoprim-sulphamethoxazole as chemoprophylaxis for pneumocystis pneumonia in renal transplant recipients. *Nephrology*.

[220] Bradley P. P., Warden G. D., Maxwell J. G., Rothstein G. (1980). Neutropenia and thrombocytopenia in renal allograft recipients treated with trimethoprim-sulfamethoxazole. *Annals of Internal Medicine*.

[221] Caluwé R., Van Laecke S., Emonds M.-P., Peeters P., Vanholder R. (2010). Immediate posttransplantation cotrimoxazole-induced immune thrombocytopenia. *American Journal of Transplantation*.

[222] Wilson J. R., Harris J. W. (1977). Hematologic side-effects of dapsone. *Ohio State Medical Journal*.

[223] Lee I., Barton T. D., Goral S. (2005). Complications related to dapsone use for Pneumocystis jirovecii pneumonia prophylaxis in solid organ transplant recipients. *American Journal of Transplantation*.

[224] Imoagene-Oyedeji A. E., Rosas S. E., Doyle A. M., Goral S., Bloom R. D. (2006). Posttransplantation anemia at 12 months in kidney recipients treated with mycophenolate mofetil: Risk factors and implications for mortality. *Journal of the American Society of Nephrology*.

[225] Pinto V., Grandy J., Zambrano P. (2008). Severe Anemia From Parvovirus B19 Infection in Pediatric Renal Transplant Recipients: Two Case Reports. *Transplantation Proceedings*.

[226] Chua M.-S., Barry C., Chen X., Salvatierra O., Sarwal M. M. (2003). Molecular profiling of anemia in acute renal allograft rejection using DNA microarrays. *American Journal of Transplantation*.

[227] Galutira P. J., Del Rio M. (2012). Understanding renal posttransplantation anemia in the pediatric population. *Pediatric Nephrology*.

[228] West L. J., Karamlou T., Dipchand A. I., Pollock-Barziv S. M., Coles J. G., McCrindle B. W. (2006). Impact on outcomes after listing and transplantation, of a strategy to accept ABO blood group-incompatible donor hearts for neonates and infants. *The Journal of Thoracic and Cardiovascular Surgery*.

[229] Newstead C. G. (2000). Lymphoproliferative disease post-renal transplantation. *Nephrology Dialysis Transplantation *.

[230] Smith E. P. (2010). Hematologic disorders after solid organ transplantation. *Hematology American Society of Hematology Education Program*.

[231] Munshi H. G., Montgomery R. B. (2000). Severe neutropenia: A diagnostic approach. *Western Journal of Medicine*.

[232] Kotton C. N., Fishman J. A. (2005). Viral infection in the renal transplant recipient. *Journal of the American Society of Nephrology*.

[233] Karras A., Thervet E., Legendre C., Groupe Coopératif de transplantation d'Ile de France (2004). Hemophagocytic syndrome in renal transplant recipients: report of 17 cases and review of literature. *Transplantation*.

[234] Ponticelli C., Alberighi O. D. C. (2009). Haemophagocytic syndrome-a life threatening complication of renal transplantation. *Nephrology Dialysis Transplantation*.

[235] Andrès E., Zimmer J., Mecili M., Weitten T., Alt M., Maloisel F. (2011). Clinical presentation and management of drug-induced agranulocytosis. *Expert Review of Hematology*.

[236] Freifeld A. G., Bow E. J., Sepkowitz K. A. (2011). Clinical practice guideline for the use of antimicrobial agents in neutropenic patients with cancer: 2010 update by the infectious diseases society of america. *Clinical Infectious Diseases*.

[237] Baldwin W. M., Larsen C. P., Fairchild R. L. (2001). Innate immune responses to transplants: A significant variable with cadaver donors. *Immunity*.

[238] Hartung T., Doecke W. D., Bundschuh D. (1999). Effect of filgrastim treatment on inflammatory cytokines and Iymphocyte functions. *Clinical Pharmacology and Therapeutics*.

[239] Von Aulock S., Boneberg E.-M., Hartung T. (2000). Intermittent G-CSF (filgrastim) treatment cannot induce lymphocytosis in volunteers. *Clinical Pharmacology & Therapeutics*.

[240] Hartung T., Döcke W.-D., Gantner F. (1995). Effect of granulocyte colony-stimulating factor treatment on ex vivo blood cytokine response in human volunteers. *Blood*.

[241] Von Aulock S., Boneberg E.-M., Diterich I., Hartung T. (2004). Granulocyte Colony-Stimulating Factor (Filgrastim) Treatment Primes for Increased ex Vivo Inducible Prostanoid Release. *The Journal of Pharmacology and Experimental Therapeutics*.

[242] Vrtovec B., Haddad F., Pham M. (2013). Granulocyte colony-stimulating factor therapy is associated with a reduced incidence of acute rejection episodes or allograft vasculopathy in heart transplant recipients. *Transplantation Proceedings*.

[243] Xu J., Aulack S. V., Lucas R., Wendel A. (2004). Potential of colony-stimulating factors to improve host defense in organ transplant recipients. *Current Opinion in Organ Transplantation*.

[244] Schmaldienst S., Bekesi G., Deicher R., Franz M., Hörl W. H., Pohanka E. (2000). Recombinant human granulocyte colony-stimulating factor after kidney transplantation: A retrospective analysis to evaluate the benefit or risk of immunostimulation. *Transplantation*.

[245] Peddi V. R., Hariharan S., Schroeder T. J., First M. R. (1996). Role of granulocyte colony stimulating factor (G-CSF) in reversing neutropenia in renal allograft recipients. *Clinical Transplantation*.

[246] Turgeon N., Hovingh G. K., Fishman J. A. (2000). Safety and efficacy of granulocyte colony-stimulating factor in kidney and liver transplant recipients. *Transplant Infectious Disease*.

[247] Tajima A., Aso Y., Kawabe K. (1991). Colony-stimulating factor for treatment of leucopenia after kidney allografting. *Transplantation Proceedings*.

[248] Colquhoun S. D., Shaked A., Jurim O., Colonna J. O., Rosove M. H., Busuttil R. W. (1993). Reversal of neutropenia with granulocyte colony-stimulating factor without precipitating liver allograft rejection. *Transplantation*.

[249] Wright H. I., Gavaler J. S., Baddour N. (1994). Granulocyte colony stimulating factor (G-CSF) combined with *α*-interferon for the treatment of liver allograft recipients with viral hepatitis. *Journal of Hepatology*.

[250] Gordon M. S., O'Donnell J. A., Mohler E. R., Cooper M. A. (1993). The use of granulocyte colony-stimulating factor in the treatment of fever and neutropenia in a heart transplant patient. *The Journal of Heart and Lung Transplantation*.

[251] Ishizone S., Makuuchi M., Kawasaki S. (1994). Effect of granulocyte colony-stimulating factor on neutropenia in liver transplant recipients with hypersplenism. *Journal of Pediatric Surgery*.

[252] Foster P. F., Mital D., Sankary H. N. (1995). The use of granulocyte colony-stimulating factor after liver transplantation. *Transplantation*.

[253] Winston D. J., Foster P. F., Somberg K. A. (1999). Randomized, placebo-controlled, double-blind, multicenter trial of efficacy and safety of granulocyte colony-stimulating factor in liver transplant recipients. *Transplantation*.

[254] Gasson J. C. (1991). Molecular physiology of granulocyte-macrophage colony-stimulating factor. *Blood*.

[255] Armitage J. O. (1998). Emerging applications of recombinant human granulocyte-macrophage colony-stimulating factor. *Blood*.

[256] Dranoff G., Crawford A. D., Sadelain M. (1994). Involvement of granulocyte-macrophage colony-stimulating factor in pulmonary homeostasis. *Science*.

[257] Metcalf D. (1986). The molecular biology and functions of the granulocyte-macrophage colony-stimulating factors. *Blood*.

[258] Hashmi A., Hussain M., Hussain Z. (1997). Use of rHu GM-CSF in renal-transplant patients developing leucopenia. *Transplantation Proceedings*.

[259] Trindade E., Maton P., Reding R. (1997). Use of granulocyte macrophage colony stimulating factor in children after orthotopic liver transplantation. *Transplantation Proceeding*.

[260] Wasler A., Iberer F., Auer T. (1995). Treatment of leukopenia with granulocyte-macrophage colony-stimulating factor after heart transplantation. *Transplantation Proceedings*.

[261] Kutsogiannis D. J., Crowther M. A., Lazarovits A. I. (1992). Granulocyte macrophage colony-stimulating factor for the therapy of cytomegalovirus and ganciclovir-induced leukopenia in a renal transplant recipient. *Transplantation*.

[262] Page B., Morin M. P., Mamzer M. F., Thervet E., Legendre C. (1994). Use of granulocyte-macrophage colony-stimulating factor in leukopenic renal transplant recipients. *Transplantation Proceedings*.

[263] Page A. V., Liles W. C. (2008). Granulocyte colony-stimulating factor, granulocyte-macrophage colony-stimulating factor, and other immunomodulatory therapies for the treatment of infectious diseases in solid organ transplant recipients. *Current Opinion in Organ Transplantation*.

[264] Crawford J., Caserta C., Roila F. (2010). ESMO Guidelines Working Group. Hematopoietic growth factors: ESMO Clinical Practice Guidelines for the applications. *Annals of Oncology*.

[265] George J. N., Raskob G. E., Shah S. R. (1998). Drug-induced thrombocytopenia: a systematic review of published case reports. *Annals of Internal Medicine*.

[266] Aster R. H., Curtis B. R., McFarland J. G., Bougie D. W. (2009). Drug-induced immune thrombocytopenia: Pathogenesis, diagnosis, and management. *Journal of Thrombosis and Haemostasis*.

[267] Hayashi M., Strouse J. J., Veltri M. A., Curtis B. R., Takemoto C. M. (2015). Immune thrombocytopenia due to Trimethoprim-Sulfamethoxazole; under-recognized adverse drug reaction in children?. *Pediatric Blood & Cancer*.

[268] Dickson H. G. (1978). Trimethoprim-sulfamethoxazole and thrombocytopenia. *Medical Journal of Australia*.

[269] Barr A. L., Whineray M. (1980). Immune Thrombocytopenia Induced by Cotrimoxazole. *Australian and New Zealand Journal of Medicine*.

[270] Papaioannides D., Bouropoulos C., Korantzopoulos P. (2003). Co-trimoxazole induced acute thrombocytopenic purpura. *Emergency Medicine Journal*.

[271] Gaydos L. A., Freireich E. J., Mantel N. (1962). The quantitative relation between platelet count and hemorrhage in patients with acute leukemia. *The New England Journal of Medicine*.

[272] Heckman K. D., Weiner G. J., Davis C. S., Strauss R. G., Jones M. P., Burns C. P. (1997). Randomized study of prophylactic platelet transfusion threshold during induction therapy for adult acute leukemia: 10,000/microL versus 20,000/microL. *Journal of Clinical Oncology*.

[273] Rebulla P., Finazzi G., Marangoni F. (1997). The threshold for prophylactic platelet transfusions in adults with acute myeloid leukemia. Gruppo Italiano Malattie Ematologiche Maligne dell'Adulto. *The New England Journal of Medicine*.

[274] Navarro J. T., Hernández J. A., Ribera J. M. (1998). Prophylactic platelet transfusion threshold during therapy for adult acute myeloid leukemia: 10, 000/microL versus 20, 000/microL. *Haematologica*.

[275] Lawrence J. B., Yomtovian R. A., Hammons T. (2009). Lowering the Prophylactic Platelet Transfusion Threshold: a Prospective Analysis. *Leukemia & Lymphoma*.

[276] Wandt H., Frank M., Ehninger G. (1998). Safety and cost effectiveness of a 10 x 109/L trigger for prophylactic platelet transfusions compared with the traditional 20 x 109/L trigger: A prospective comparative trial in 105 patients with acute myeloid leukemia. *Blood*.

[277] Bishop J. F., Schiffer C. A., Aisner J., Matthews J. P., Wiernik P. H. (1987). Surgery in acute leukemia: A review of 167 operations in thrombocytopenic patients. *American Journal of Hematology*.

[278] British Committee for Standards in Haematology Blood Transfusion Task Force (2003). Guidelines for the use of platelet transfusions. *British Journal of Haematology*.

[279] Schiffer C. A., Anderson K. C., Bennett C. L. (2001). Platelet transfusion for patients with cancer: Clinical practice guidelines of the American Society of Clinical Oncology. *Journal of Clinical Oncology*.

[280] Vavricka S. R., Walter R. B., Irani S., Halter J., Schanz U. (2003). Safety of lumbar puncture for adults with acute leukemia and restrictive prophylactic platelet transfusion. *Annals of Hematology*.

[281] Brachemi S., Bollee G. (2014). Renal biopsy practice: What is the gold standard?. *World Journal of Nephrology*.

[282] Webert K. E., Cook R. J., Sigouin C. S., Rebulla P., Heddle N. M. (2006). The risk of bleeding in thrombocytopenic patients with acute myeloid leukemia. *Haematologica*.

[283] Slichter S. J., Davis K., Enright H. (2005). Factors affecting posttransfusion platelet increments, platelet refractoriness, and platelet transfusion intervals in thrombocytopenic patients. *Blood*.

[284] Bowden R. A. (1995). Transfusion-transmitted cytomegalovirus infection. *Immunological Investigations*.

[285] Kuter D. J., Bussel J. B., Lyons R. M. (2008). Efficacy of romiplostim in patients with chronic immune thrombocytopenic purpura: a double-blind randomised controlled trial. *Lancet*.

[286] Bussel J. B., Cheng G., Saleh M. N. (2007). Eltrombopag for the treatment of chronic idiopathic thrombocytopenic purpura. *The New England Journal of Medicine*.

[287] McHutchison J. G., Dusheiko G., Shiffman M. L. (2007). Eltrombopag for thrombocytopenia in patients with cirrhosis associated with hepatitis C. *The New England Journal of Medicine*.

[288] Olnes M. J., Scheinberg P., Calvo K. R. (2012). Eltrombopag and improved hematopoiesis in refractory aplastic anemia. *The New England Journal of Medicine*.

[289] Parameswaran R., Lunning M., Mantha S. (2014). Romiplostim for management of chemotherapy-induced thrombocytopenia. *Supportive Care in Cancer*.

[290] Kuter D. J. (2015). Managing thrombocytopenia associated with cancer chemotherapy. *Oncology (Williston Park)*.

[291] Winer E. S., Safran H., Karaszewska B. (2015). Eltrombopag with gemcitabine-based chemotherapy in patients with advanced solid tumors: A randomized phase i study. *Cancer Medicine*.

[292] Chawla S. P., Staddon A., Hendifar A., Messam C. A., Patwardhan R., Kamel Y. M. (2013). Results of a phase I dose escalation study of eltrombopag in patients with advanced soft tissue sarcoma receiving doxorubicin and ifosfamide. *BMC Cancer*.

[293] Mathew A., Woytowitz D., Smith R. E., Mehta R. P. (2013). Tacrolimus-Induced Refractory Immune Thrombocytopenia In Solid Organ Transplant Patients. *Blood*.

[294] Grenda R., Jarmużek W., Latoszyńska J., Prokurat S., Rubik J. (2016). Eltrombopag (thrombopoietin-receptor agonist) and plasmapheresis as rescue therapy of acute post-renal transplant immune thrombocytopenia in a child with Schimke immuno-osseous dysplasia—case report. *Pediatric Transplantation*.

